# Supplementary material for: Prospective study of change in liver function and fat in patients with colorectal liver metastases undergoing preoperative chemotherapy: protocol for the CLiFF Study
Source: BMJ Open. 2020 Sep 23;10(9):e027630. doi: 10.1136/bmjopen-2018-027630 (PMC7513559; doi:10.1136/bmjopen-2018-027630)
Supplement: Supplementary data [file bmjopen-2018-027630supp001.pdf]

APPENDIX 1 - STUDY SCHEDULE

				Standard care assessments		Experimental added assessments		
**Timeline (weeks)**		**Oncology Outpatient Department**	**Blood Tests**	**CT**	**Histology**	**LiMAx**	**MR**	**EQ-5D**
**BASELINE**		**Patient identification HPB MDT/ Oncology**		✓				
		**New Patient Oncology Clinic – CLiFF Study information**	✓					
**1**	Day 1	Oncology Clinic – Assessment, Bloods	✓			✓	✓	✓
	Day 2	**Chemotherapy- Cycle 1**						
**3**	Day 1	Oncology Clinic – Assessment, Bloods	✓					
	Day 2	**Chemotherapy- Cycle 2**						
**5**	Day 1	Oncology Clinic – Assessment, Bloods	✓					
	Day 2	**Chemotherapy- Cycle 3**						
**7**	Day 1	Oncology Clinic – Assessment, Bloods	✓			✓		
	Day 2	**Chemotherapy- Cycle 4**						
**9**	Day 1	Oncology Clinic – Assessment, Bloods	✓					
	Day 2	**Chemotherapy- Cycle 5**						
**11**	Day 1	Oncology Clinic – Assessment, Bloods	✓					
	Day 2	**Chemotherapy- Cycle 6**						
**13**	*Chemo finished	Oncology Clinic – Final Assessment, Bloods **ONCOLOGY DISCHARGE**	✓	✓		✓	✓	✓
**19**	**Post-Chemotherapy Week 8**							
	**Additional Visit to Christie Site** LiMAx and MR					✓	✓	✓
**Pre-Op** (~ Wk 19/20)		Pre-operative assessment clinic at Manchester Foundation Trust HPB Dept	✓					
**6 weeks post-op** (~ Wk 26)		Post-operative review clinic at Manchester Foundation Trust HPB Dept	✓		✓			✓

APPENDIX 2 – STUDY ASSESSMENTS

	Event	Baseline	Pre-chemo	2nd cycle	3rd cycle	4th cycle	5th cycle	6th cycle	Following last cycle of chemotherapy	Approx 8 wks post-chemo/ pre-op assessment	Post-op Assessment
**Standard Care Events/ Assessments**	History	✓									
	Physical examination	✓	✓	✓	✓	✓	✓	✓	✓	✓	✓
	Height	✓									
	Weight	✓	✓	✓	✓	✓	✓	✓	✓	✓	✓
	WHO perf score	✓	✓	✓	✓	✓	✓	✓	✓	✓	✓
	FBC	✓	✓	✓	✓	✓	✓	✓	✓	✓	✓
	Biochemistry [b]	✓	✓	✓	✓	✓	✓	✓	✓	✓	✓
	CEA	✓								✓	
	Coagulation [c]	✓	✓	✓	✓	✓	✓	✓	✓	✓	
	Pregnancy test [d]	✓									
	CT	✓							✓		
	Histopathology										✓
**Experimental Additional Events/ Assessments**	Informed Consent		✓								
	EQ-5D Score		✓						✓	✓	✓
	MR		✓						✓	✓	
	LiMAx		✓			✓			✓	✓	

Parmar KL, *et al. BMJ Open* 2020; 10:e027630. doi: 10.1136/bmjopen-2018-027630
