# Supplementary material for: Prospective study of change in liver function and fat in patients with colorectal liver metastases undergoing preoperative chemotherapy: protocol for the CLiFF Study
Source: BMJ Open. 2020 Sep 23;10(9):e027630. doi: 10.1136/bmjopen-2018-027630 (PMC7513559; doi:10.1136/bmjopen-2018-027630)
Supplement: Author's manuscript [file bmjopen-2018-027630.draft_revisions.pdf]

BMJ Open is committed to open peer review. As part of this commitment we make the peer review history of every article we publish publicly available.

When an article is published we post the peer reviewers' comments and the authors' responses online. We also post the versions of the paper that were used during peer review. These are the versions that the peer review comments apply to.

The versions of the paper that follow are the versions that were submitted during the peer review process. They are not the versions of record or the final published versions. They should not be cited or distributed as the published version of this manuscript.

BMJ Open is an open access journal and the full, final, typeset and author-corrected version of record of the manuscript is available on our site with no access controls, subscription charges or pay-per-view fees (http://bmjopen.bmj.com).

If you have any questions on BMJ Open's open peer review process please email info.bmjopen@bmj.com

BMJ Open

A prospective study of Change in Liver Function and Fat in patients with colorectal liver metastases undergoing pre-operative chemotherapy: the CLiFF study

Journal:	*BMJ Open*
Manuscript ID	bmjopen-2018-027630
Article Type:	Protocol
Date Submitted by the Author:	01-Nov-2018
Complete List of Authors:	Parmar, Kat; University of Manchester, Division of Cancer Sciences, School of Medical Sciences, Faculty of Biology, Medicine and Health; Manchester Cancer Research Centre<br>O'Reilly, Derek; Central Manchester University Hospitals NHS Foundation Trust, Hepatobiliary Surgery<br>Valle, Juan; Christie NHS Foundation Trust, Oncology; University of Manchester, Division of Cancer Sciences, School of Medical Sciences, Faculty of Biology, Medicine and Health<br>Braun, Michael; Christie NHS Foundation Trust, Oncology<br>Naish, Jo; University of Manchester Institute of Cardiovascular Sciences<br>Williams, Steve; University of Manchester Centre for Imaging Sciences<br>Malcomson, Lee; University of Manchester, Division of Cancer Sciences, School of Medical Sciences, Faculty of Biology, Medicine and Health; Christie NHS Foundation Trust<br>Cresswell, Katharine; Manchester University NHS Foundation Trust, Public Programmes Team, Research and Innovation Division<br>Bamford, Colin; NIHR Manchester Biomedical Research Centre, Cancer Patient and Public Advisory Group<br>Renehan, Andrew; University of Manchester, 1 Division of Cancer Sciences, School of Medical Sciences, Faculty of Biology, Medicine and Health; Christie NHS Foundation Trust
Keywords:	Colorectal surgery < SURGERY, Hepatobiliary surgery < SURGERY, CHEMOTHERAPY

SCHOLARONE™
Manuscripts

Type of article: study protocol (formatted for BMJ Open)

A prospective study of Change in Liver Function and Fat in patients with colorectal liver metastases undergoing pre-operative chemotherapy: the CLiFF study

Kat L Parmar,[1,2,3,4] Derek O'Reilly,[4] Juan W Valle,[1,3] Michael Braun,[3] Jo H Naish,[5] Steve R Williams,[6] Lee Malcomson,[1,2,3] Katharine Cresswell,[2,7,8] Colin Bamford,[9] Andrew G Renehan[1,2,3]

[1] Division of Cancer Sciences, School of Medical Sciences, Faculty of Biology, Medicine and Health, University of Manchester

[2] Manchester Cancer Research Centre, NIHR Manchester Biomedical Research Centre, Manchester

[3] The Christie NHS Foundation Trust, Manchester

[4] Department of Hepatobiliary Surgery, Manchester University Foundation Hospitals, Manchester

[5] Division of Cardiovascular Sciences, School of Medical Science, Faculty of Biology, Medicine and Health, University of Manchester

[6] Centre for Imaging Sciences, School of Health Sciences, Faculty of Biology, Medicine and Health, University of Manchester

[7] Division of Evolution and Genomic Sciences, School of Biological Sciences, Faculty of Biology, Medicine and Health, University of Manchester

[8] Public Programmes Team, Research and Innovation Division, Manchester University NHS Foundation Trust, Manchester

[9] NIHR Manchester Biomedical Research Centre Cancer Patient and Public Advisory Group

Running title: *Liver fat and function after chemotherapy*

Keywords: liver injury, liver steatosis, liver resection, colorectal cancer metastases, chemotherapy

Correspondence to:

Miss Kat Parmar
Division of Cancer Sciences, School of Medical Sciences,
Faculty of Biology, Medicine and Health,
University of Manchester,
The Christie NHS Foundation Trust,
Wilmslow Road
Manchester,
M20 4BX UK
Tel: +44 161 3060870
E-mail: kat.parmar@manchester.ac.uk

Abstract: 266 words (max: 300); main text: 3604 words (max: 4000); x2 figures; 105 references supplemental material (2 pages); language: UK English.

For peer review only

ABSTRACT

Introduction

Pre-operative chemotherapy in patients undergoing resection for colorectal liver metastases (CLM) improves oncological outcomes. However, chemotherapy-associated liver injury (occurring in two patterns: vascular and fat deposition) is a real clinical concern prior to hepatic resection. After major liver resection, regeneration of the residual liver is a prerequisite for recovery and avoidance of liver failure, but this regenerative capacity may be hindered by chemotherapy. Thus, there is a need to predict for this serious complication. Over the past two decades, several tests and derived indices have been developed, which have failed to achieve clinical utility, mainly as they were indirect measurements of liver function. Here, we will use a novel test of liver function (the LiMAx test), and measure liver fat using magnetic resonance (MR) imaging.

Methods and analysis

This prospective study will assess changes in liver function longitudinally, measured by the LiMAx test, and liver fat, measured by advanced MR imaging using both MR spectroscopy and the modified Dixon method, in up to 35 patients undergoing pre-operative chemotherapy for CLM. The primary outcomes will be the changes in liver function and fat compared to baseline pre-chemotherapy measurements. Secondary outcome measures include: routinely measured liver function blood tests, anthropometric measurements, post-operative histology and digital quantification of fat, post-operative complications and mortality and quality of life.

Ethics and dissemination

The study was approved by a National Health Service Research Ethics Committee and registered with the Health Research Authority. Dissemination will be via international and national conferences, and the NIHR network. Manuscripts will be published.

Trial Registration Number:

This study is registered online at www.clinicaltrials.gov (registration number NCT03562234).

For peer review only

Strengths and limitations of this study

- This is an evaluation of the effects of pre-operative chemotherapy upon liver function and fat using novel techniques, addressing the important clinical issue of liver injury prior to hepatic resection
- These non-invasive techniques permit repeated measurements to assess change in liver function and fat over time, permitting interpretation of causality – this has not been possible in the past
- Chemotherapy-related changes in liver fat and function will be assessed for reversibility and correlated with both standard assessment measures and post-operative histology, providing insight into the scale of the injuries prior to the post-chemotherapy recovery period
- Changes and recovery in liver fat and function will be correlated with post-operative morbidity and mortality
- A potential limitation is that this is a single-site observational study with a relatively small sample size

For peer review only

Background

Colorectal cancer (CRC) is the second most common cause of cancer death in the UK.[1] Death is usually due to metastatic disease, with the most common site being the liver (colorectal liver metastases - CLM). There is an incidence of 15-20% CLM at initial diagnosis of CRC[2] with up to 50% of patients ultimately developing CLM.[3-5 6]

Surgical resection of CLM is the only single-modality therapy associated with cure and remains the mainstay of treatment.[7] The resectability of CLM depends upon the volume and function of the future liver remnant (FLR). Volume alone is not an ideal assessment of liver functionality as it cannot reliably predict outcome.[7-11 12] FLR function is decreased in patients with parenchymal disease, despite being of equal volume to patients with heathy livers,[13-15] and liver disease may result in impaired liver regeneration.[7 11 16-18] In the presence of impaired liver function the surgical strategy may need to be adapted, either to increase the volume of the FLR or perform a less extensive resection.[2 19] Accurate pre-operative assessment of liver function is therefore crucial.[20]

With best supportive care, the median survival of patients with CLMs is 6-13 months (where five-year survival is rare).[21] In patients fit enough to receive combined hepatic resection and chemotherapy, the 5-year survival improves to 50%.[22 23 24 24-27] Pre-operative chemotherapy is used in the setting of both resectable and unresectable CLM, with the intentions of (i) improving survival (neoadjuvant approach) in operable CLM; and (ii) converting inoperable CLM into operable (downsizing approach). Approximately 70% of patients presenting with CLM are initially deemed unresectable,[28] and after systemic chemotherapy, up to 40% of patients may be judged resectable.[61 29 30-33] Outcomes in these "downsized" cases can be similar to those initially presenting with resectable disease.[34 35] In the setting of operable CLM, the randomized EPOC trial reported a 7.3% improvement in 3-year progression-free survival with addition of chemotherapy versus resection alone for CLM, and highlighted the

For peer review only

issue that organ-specific resectional surgery does not address the systemic process, leading to high post-operative recurrence rates.[36]

The main concern regarding the pre-operative administration of chemotherapy for CLM is direct pathologic changes to the liver, known as chemotherapy-associated liver injury (CALI). CALIs comprise two main types of liver injury: vascular changes and fatty changes. These injuries are not mutually exclusive; patients who have developed fatty changes can simultaneously develop vascular changes.[7] There are many inconsistent reports on the types of liver injury seen and their associations with specific chemotherapy regimens.[11 30 36-57] There is considerable heterogeneity in the methods used to assess changes to the liver parenchyma. Earlier studies used CT scans and liver biopsies[37-40] and later studies used post-operative histology, reported using a variety of scoring systems.[11 30 36 41-57] Radiation exposure resulting from CT scans and the invasive nature of liver biopsies mean that neither technique is suited to providing repeated measurements, and post-operative specimens provide only a one-off assessment of the liver parenchyma. None of these assessment tools are therefore ideally suited to measuring change over time.

The link between CALIs and post-operative morbidity and mortality remains unclear with reports to date being mixed.[30 36 41-44 46-52 54-58] Some studies such as the one by Karoui et al[46] have demonstrated that CALIs increased the risk of post-operative liver failure (11% v 0%), with others such as Vauthey et al[47] demonstrating increased post-operative mortality (increased 90-day mortality in patients with steatohepatitis 14.7% v 1.6%, P=0.001; steatohepatitis associated with pre-operative irinotecan 20.2% v 4.4%, P<0.001).

Post-operative morbidity and mortality are often related to inadequate function of the remnant liver, resulting in post-operative liver failure - an important cause of mortality after partial liver resection.[14 59 60] A meta-analysis estimated the overall incidence of liver failure after hepatectomy to lie between 0.7% and 9.1%,[61] with most recent studies reporting liver

failure rates within this range.[62 57] The improved detection of CALIs, assessment of their impact upon post-operative outcomes and pre-operative assessment of the FLR are thus important clinical issues. Several tests and derived indices are in use, including conventional blood parameters of liver function (liver enzymes, albumin, bilirubin, INR), Child-Pugh Classification, scintigraphy, galactose elimination test and indocyanine green (ICG) clearance rate. These assessments are suboptimal as they comprise indirect measurements of liver function.

Advances in chemotherapy and hepatic surgery have expanded the pool of candidates for potentially curative hepatic resection for CLM,[7] many of whom will undergo pre-operative chemotherapy. Recognition of CALIs emphasises the need to fully assess each patient and utilise individualised planning, giving attention to the planned extent of resection, the choice of chemotherapy regimen and the potential consequences upon the hepatic parenchyma. Future challenges include the refinement of liver function assessment and the establishment of better methods for evaluation and diagnosis of post-chemotherapy liver injury.[7]

This study aims to address these challenges by utilising the following two novel techniques for assessing liver function and liver fat, measuring these repeatedly before after chemotherapy, and correlating these changes to post-operative outcomes. Performing repeated measurements over time of both liver fat deposition and liver function during and after the cessation of chemotherapy will permit better interpretation of causality and potential reversibility. This will address the mechanistic question shown in **Figure 1**: is chemotherapy-induced hepatic fat accumulation driven through liver injury as shown in Hypothesis A, or unrelated as in Hypothesis B?

For peer review only

Figure 1 (See separate image file)

Understanding these relationships and making use of the observation that pre-operative chemotherapy is a model of an extreme accelerated phenotype of liver fat deposition may also help to address some of the underlying mechanistic questions in obesity-related cancers.

The LiMAx test

The Liver Maximum capacity (LiMAx) test was developed as a novel clinical evaluation of liver function, aimed at overcoming the difficulties in accurately assessing pre-operative liver function prior to hepatic resection.[63] It is based on the metabolism of a ^{13}C-labelled substrate by the hepatocyte-specific enzyme system P450 CYP1A2, the activity of which is not influenced by drugs or genetic variations,[64] is distributed ubiquitously throughout the liver,[65] and shows a clear discrimination between normal and abnormal liver function independent of cholestasis.[66] The intravenous administration of ^{13}C-methacetin results in a significant alteration of the normal expired breath $^{13}CO_2$:$^{12}CO_2$ ratio[67] detected by breath analysis performed by an infrared spectroscopy-based FLIP device.

The LiMAx test was initially used in pre- and post-operative hepatectomy assessment, where resection of a specific percentage of functional liver volume was found to lead to an equivalent reduction in LiMAx value.[68] The LiMAx test was found to represent an accurate surrogate of liver function capacity,[68] to be an accurate predictor of post-operative liver failure and mortality,[68 63 69 70] and to be unaffected by age and gender.[71]

The LiMAx test has been evaluated as a tool with which to diagnose CALIs. LiMAx was found to be superior to other methods of assessing liver function both in detecting CALIs and their resolution, when compared to standard biochemical liver parameters (INR, bilirubin,

For peer review only

aspartate transaminase) and ICG-clearance rates.[72 73] However, it has never been used repeatedly in the setting of CLM or correlated with post-operative outcomes.

Advanced MR imaging

Traditional assessment methods failed to provide an ideal measurement tool for liver fat content (hepatic fat fraction – HFF). Ultrasonography scan (USS) and computed tomography (CT) have the ability to demonstrate fatty infiltration, however, they are unable to quantify HFF.[74-77] USS is preferred for qualitative assessment because CT sensitivity is time-dependent and protocol specific[78] and is not suitable for use in longitudinal monitoring due to its reliance on ionising radiation.[79] On the other hand, USS is low-cost, safe, and readily accessible.[79] However, USS has limited repeatability and reproducibility, and the outputs have only modest correlation with biopsy results.[78 80] Liver biopsy provides a quantitative method for the assessment of fatty liver disease, however, this is an invasive procedure hampered by sampling errors as fatty infiltrations can be unevenly distributed throughout the liver,[81-83] significant inter-observer variability[84] and potential complications such as bleeding and mortality.[85]

Magnetic resonance (MR) imaging using standard pulse sequences is insensitive to diffuse fatty infiltrations.[86-88] Techniques have been developed with the aim of further exploiting the differences in resonance frequencies between water and fat proton signals in order to assess HFF with increased accuracy, including magnetic resonance spectroscopy (MRS)[89] and the modified Dixon technique (mDixon).[90]

MRS utilises a much larger sample of the liver as compared to a typical biopsy sample, minimising the chance of sampling error, and is sensitive enough to detect small amounts of fat that may be histologically undetectable.[91] It is considered to be the optimal non-invasive method for the accurate assessment of liver fat,[92] being the technique shown to produce the most reliable and reproducible measurements.[79 81 91 93-97 98] MRS has been validated as an

For peer review only

accurate technique against the histological evaluation of fatty liver changes in multiple studies.[81 93-96 99 100] However, MRS has not achieved widespread clinical use despite the undisputed strengths of this technique. This may be due to poor availability of expertise and equipment outside the research setting.[90]

Techniques of quantitative magnetic resonance imaging have recently been introduced for the acquisition of proton density fat fraction maps covering large portions of the liver. These modified Dixon techniques (mDixon) show promising results in assessing HFF in comparisons with histological scoring systems[101-103] and correlate well with MRS.[90]

The Manchester Obesity and Cancer Research Group run a long-term research programme developing non-invasive imaging techniques to quantify liver fat. We have previously validated the use of MRS and the mDixon method against digital histological determination of fatty infiltration in post-surgical specimens, quantifying and characterising fat deposition in patients with CLM undergoing resection.[104 105] These previous studies were cross-sectional whereas the current study will evaluate changes over time in order to better assess their relationship to chemotherapy and their potential reversibility.

AIMS

To prospectively assess the changes in liver function and fat resulting from the administration of pre-operative chemotherapy prior to hepatic resection for colorectal liver metastases (Change in Liver Function and Fat – the CLiFF study). These changes will be assessed repeatedly using the novel techniques of the LiMAx test and advanced MR imaging as demonstrated in **Figure 2**, then correlated with standard assessments and post-operative outcomes. To our knowledge, there is no prospective evaluation of this type to-date.

Figure 2 – Study Participant Timeline (See separate image file)

For peer review only

Primary outcome measures

1. Change in liver function measured by the LiMAx test performed using the FLIP device, comparing baseline measurements with measurements during and after chemotherapy.
2. Change in liver fat as a percentage (Hepatic Fat Fraction – HFF) measured by advanced Magnetic Resonance imaging (utilising both Magnetic Resonance Spectroscopy and the modified Dixon method), comparing baseline measurements with measurements during and after chemotherapy.

Secondary outcome measures

1) Changes in routinely collected liver enzymes by blood testing
2) Digital histological quantification of intrahepatic fat
3) Correlations with BMI and image-determined anthropometric measurements e.g. subcutaneous adipose tissue (SAT), visceral adipose tissue volumes (VAT) and muscle mass
4) Grade of steatosis scores (NAS-CRN) from routine histology reporting
5) Post-operative outcomes, including peri-operative mortality and morbidity (Clavien-Dindo classification, Comprehensive Complication Index, International Study Group for Liver Surgery complications).
6) Change in health-related quality of life by measurement of EQ-5D score

METHODS

Study design

This is a non-randomised prospective observational study of up to 35 patients with CLM.

Study setting

The study is sponsored by the University of Manchester and will be performed at The Christie NHS Foundation Trust and the University of Manchester Wolfson Molecular Imaging

For peer review only

Centre. Participant identification will take place at the joint Christie-Manchester Foundation Trust hepatopancreatobiliary (HPB) multidisciplinary team (MDT) meeting. Team members will screen all patients for whom an MDT decision is made that a course of pre-operative chemotherapy is indicated prior to potentially curative hepatic resection of CLM.

Institutions

From October 6, 2014, a single Improving Outcomes Guidance-compliant HPB Service has been located at the Manchester Royal Infirmary site at Manchester University NHS Foundation Trust. It hosts the regional specialist HPB MDT meeting. All patients selected to undergo neoadjuvant chemotherapy prior to hepatic resection from this MDT will receive this treatment via the oncology service delivered from the Christie Hospital.

The Wolfson Molecular Imaging Centre is part of the University of Manchester. This centre provides state-of-the art imaging facilities and radiological expertise in the advanced imaging techniques of MRS and mDixon. The Philips 1.5T scanner at WMIC was upgraded to provide mDixon in addition to the established MRS.

Investigators

The CLiFF study is led by a dedicated clinical research fellow, as part of a University-registered thesis. The supervisory team provide the relevant expertise in surgical cancer research, hepatobiliary surgery, medical oncology and imaging science, with additional research and statistical support available as required.

Inclusion criteria

Patients with liver metastases of colorectal origin, undergoing pre-operative standard chemotherapy, with the following criteria:

1. Histologically verified adenocarcinoma of the colon or rectum with radiological evidence of potentially resectable liver metastases;

For peer review only

2. No evidence of unresectable non-hepatic metastatic disease;
3. Adequate haematological and hepatic function defined by: Hb ≥ 10g/dl, WBC ≥ 3.0 x 10^9/L, ANC ≥ 1.5 x 10^9/L, platelet count ≥ 100,000/ mm^3, total bilirubin < 30 μmol/L, serum AST, ALT and alkaline phosphatase ≤ 5 x upper limit of normal;
4. Written informed consent and able to attend long-term follow-up.

Exclusion criteria

1. Presence of a medical or psychiatric condition that impairs their ability to give informed consent
2. Presence of any other serious uncontrolled medical conditions
3. Evidence of unresectable non-hepatic metastatic disease

Patient identification

Patients will be screened for inclusion initially via discussion at the specialist HPB MDT meeting and by referral to the Medical Oncology service. Subsequent to an MDT decision that pre-operative chemotherapy is indicated prior to potentially curative resection of colorectal liver metastases, the CLiFF study team will screen for the remaining inclusion criteria. Potential participants will be invited to join the study via the provision of verbal and written information regarding the CLiFF study. Confirmation of the wish to participate will subsequently be confirmed and informed consent taken prior to beginning chemotherapy.

Data collection

Participants will undergo assessment of liver function by LiMAx testing and HFF by advanced MR imaging at the times outlined in the study schedule shown in Appendix 1 and 2. All clinicians involved in the medical and surgical treatment of the study participants will be blinded to the results of the LiMAx testing and MR imaging. The study MR images will not be

For peer review only

reported by a clinical radiologist. All other aspects of treatment will remain unchanged and proceed as per standard care.

Data collection will include LiMAx tests and MR results, in addition to the results from all other assessments outlined in Appendix 2. The LiMAx tests and MR scans will be performed directly by the clinical research fellow. The remaining information will be taken from the medical notes, comprising the results from routine oncology outpatient assessments, the post-operative histology report and the post-operative outcomes following hepatic resection. Informed consent will be taken for the research team to access this information from the medical records.

Data management

The NHS code of confidentiality will be observed, with only the clinical care team and team directly involved in the research having access to any identifiable data. All data will be pseudonymised with a unique identifier and stored in a secure encrypted database. A key to patient identifiers will be stored in a separate encrypted document. Data stored by the research team will therefore not contain patient identifiers. Data will be collected and stored in accordance with the General Data Protection Regulation (GDPR) and Data Protection Act 2018. The University of Manchester, as Data Controller for this study, takes responsibility for the protection of any personal information collected by this study. All researchers will be appropriately trained in data protection.

Statistical analysis & power calculation

Our previous work reported a mean (±sd) Hepatic Fat Fraction of 4 (±2) %.[105] Following chemotherapy, we estimate at least a 50% increase in HFF i.e. increase to mean (sd) = 6 ± 2.5%. We have planned a recruitment of at least 35 patients. As attrition (for multiple reasons) is common in this type of study, of the 35 patients, we conservatively estimate that

For peer review only

25 patients will have complete data from at least two MR evaluations. The resultant power is 98% (alpha: 0.05; within-person correlation: 0.7).

For the LiMAx testing, which is more frequent in the study schedule, we estimate at least 32 patients (10% attrition rate) will have complete data from two LiMAx tests. Based on the Lock et al [2017] study in patients undergoing pre-resection chemotherapy[73] [mean (±sd): 340 ± 95 vs. 391 ± 82 μg/kg/h], the resultant power is 84% (alpha: 0.05; within-person correlation: 0.7).

Statistical support will be provided where required by the University of Manchester Cancer Data Science Team. The main analysis will focus on intra-patient change in both liver function (measured by the LiMAx test) and hepatic fat fraction (measured by advanced MR imaging) which occurs subsequent to chemotherapy, and their recovery prior to surgery. These changes will be related to subsequent clinical course, CT volumetry and histopathological analysis of the resected liver. We will explore the use of ANOVA methods and mixed-effects methods to account for within-person correlations. For each time point, standard approaches to categorical (chi-squared test) and continuous (Mann-Whitney U test) variables will be used. Correlation matrices will be constructed to assess relationships between LiMAx and HFF with other continuous variables using Spearman's Rank Correlation Coefficient.

To account for multiple testing, a p-value of <0.01 will be considered to indicate statistical significance. Predictors of HFF at each time point will be explored using multivariable linear regression models. To reduce the anticipated right skewness of the distributions, logarithmic transformation will be explored.

Because of the large number of variables and the anticipated high levels of correlations, we will use factor-cluster methods. There will be at least five clusters: patient-related factors;

For peer review only

routine blood measures; CT-derived anthropometrics; LiMAx readouts; and MR readouts. Separate models will be developed for each cluster and significant ($p < 0.05$) variables selected for the final model.

Anticipated recruitment

At the specialist HPB MDT, there are approximately 120 resections per year performed for CLM. Approximately half these patients will receive pre-operative chemotherapy. Of these 60 patients, 40 will have their chemotherapy delivered at the oncology centre and will be potentially eligible for recruitment. Over the past 4 years, the numbers of liver resections performed for CLM has consistently increased year on year. We therefore anticipate that the recruitment target of 35 patients to be recruited and followed up within two years is achievable.

Quality assurance

The quality of this study has been assessed by the following means:

- Departmental review within the University of Manchester
- Peer review by professionals with relevant expertise (Clinical trialists, statisticians, surgeons, oncologists and imaging scientists)
- Review by the Research Practice Governance Team at the University of Manchester (Sponsor Institution) and the Research & Development team at the Christie NHS Foundation Trust (host)
- Peer review arranged by the North West Innovation Service as part of a successful competitive funding application
- Review by the Royal College of Surgeons research department as part of a successful competitive application for a research fellowship

For peer review only

- Independent peer review as part of the registration process for ClinicalTrials.gov, a publicly accessible database of worldwide research studies, maintained by the National Library of Medicine at the National Institutes for Health (US) (NCT03562234)

Timetable

Time Period	Date
Study Set-Up and Approvals	01/04/2018 - 19/06/2018
Participant Recruitment	01/09/2018 - 01/08/2019
Data collection	01/09/2019- 01/12/2019
Data Analysis and Write-Up	01/01/2020 – 31/03/2020

ETHICS AND DISSEMINATION

Ethical approval

Ethical approval for this study was granted by the National Health Service North West Research Ethics Committee following the meeting held on 27th July 2018 (REC Reference 18/NW/0531).

Registration

This study was registered, reviewed and approved by the Health Research Authority (HRA). It has been registered with the sponsor institution (University of Manchester) and approved by their Research Practice Governance Team. The host institution (Christie NHS Foundation Trust) Research & Development team have issued approval of their capability and capacity to host this study. The study was registered online at www.clinicaltrials.gov, registration number NCT03562234.

Patient and Public Involvement

For peer review only

The Public Programmes Team at the Manchester University NHS Foundation Trust hosted a discussion group for the CLiFF Study with six members of the NIHR Manchester Biomedical Research Centre funded Cancer Research Advisory Panel on 24/05/2018. The six attending members were all patients/carers with personal experience of CLM treatment. This event was attended by the Principle Investigator to actively seek and hear the views of the patient and public representatives. The group unanimously found that this area of research was important, found no feasibility or acceptability issues with the study and thought it likely that they would take part if asked. The following modifications were made in response to the session:

1) **Amendments to Participant Information Sheet:**

   All group suggestions were incorporated, including the requested addition of a clearly highlighted opening statement explaining that treatment would not be modified as the result of participating in this study.

2) **Training to use long-term intravenous access devices:**

   Patients who had previously undergone pre-operative chemotherapy for CLM expressed frustration that whilst many of them had long-term intravenous access devices for the duration of their treatment, inadequate training to access these devices resulted in additional exposure to needles. They requested that where possible, these long-term devices be used for the intravenous injection component of the LiMAx test. In response to this request, the Principal Investigator underwent additional training to ensure that long-term intravenous access devices could be utilised for the LiMAx tests performed in this study.

One patient member of the Cancer Research Advisory Panel contributed substantially and subsequently became a co-author of this protocol paper (CB).

Dissemination

For peer review only

This study will be submitted for presentation at national or international surgical conferences. Manuscript(s) will be prepared following close of the study.

Figure Legends

Figure 1: Direct Acyclic Graph

Figure 2: Study Participant Timeline

Authorship contributions

The study concept was conceived by JWV, DOR and AGR. The present protocol was written by DOR, AGR and KLP. All authors contributed to the revision and preparation of the manuscript.

This research has received funding from the following sources:

Humedics GMBH; National Institute for Health Research Manchester Biomedical Research Centre; North West NHS Innovation service; The Christie NHS Foundation Trust charitable funds; The Royal College of Surgeons of England Fellowship scheme

Conflicts of interest: AGR reports personal fees from Merck Serona, personal fees from Janssen-Cilag, grants and personal fees from Sanofi Pasteur MSD, outside the submitted work. The remaining authors declare no conflicts.

For peer review only

References

1. CRUK. Cancer Research UK - Bowel Cancer Statistics 2018 [Available from: http://www.cancerresearchuk.org/health-professional/cancer-statistics/statistics-by-cancer-type/bowel-cancer accessed 2/04/2018.
2. Fiorentini G, Sarti D, Aliberti C, et al. Multidisciplinary approach of colorectal cancer liver metastases. *World J Clin Oncol* 2017;8(3):190-202. doi: 10.5306/wjco.v8.i3.190 [published Online First: 2017/06/24]
3. Manfredi S, Lepage C, Hatem C, et al. Epidemiology and management of liver metastases from colorectal cancer. *Ann Surg* 2006;244(2):254-9. doi: 10.1097/01.sla.0000217629.94941.cf [published Online First: 2006/07/22]
4. Nordlinger B, Van Cutsem E, Gruenberger T, et al. Combination of surgery and chemotherapy and the role of targeted agents in the treatment of patients with colorectal liver metastases: recommendations from an expert panel. *Ann Oncol* 2009;20(6):985-92. doi: 10.1093/annonc/mdn735 [published Online First: 2009/01/21]
5. Adam R, De Gramont A, Figueras J, et al. The oncosurgery approach to managing liver metastases from colorectal cancer: a multidisciplinary international consensus. *Oncologist* 2012;17(10):1225-39. doi: 10.1634/theoncologist.2012-0121 [published Online First: 2012/09/11]
6. Renehan AG, Egger M, Saunders MP, et al. Impact on survival of intensive follow up after curative resection for colorectal cancer: systematic review and meta-analysis of randomised trials. *BMJ* 2002;324(7341):813. [published Online First: 2002/04/06]
7. Zorzi D, Laurent A, Pawlik TM, et al. Chemotherapy-associated hepatotoxicity and surgery for colorectal liver metastases. *Br J Surg* 2007;94(3):274-86. doi: 10.1002/bjs.5719 [published Online First: 2007/02/23]
8. Schroeder RA, Marroquin CE, Bute BP, et al. Predictive indices of morbidity and mortality after liver resection. *Ann Surg* 2006;243(3):373-9. doi: 10.1097/01.sla.0000201483.95911.08 [published Online First: 2006/02/24]
9. Dinant S, de Graaf W, Verwer BJ, et al. Risk assessment of posthepatectomy liver failure using hepatobiliary scintigraphy and CT volumetry. *J Nucl Med* 2007;48(5):685-92. doi: 10.2967/jnumed.106.038430 [published Online First: 2007/05/04]
10. Erdogan D, Heijnen BH, Bennink RJ, et al. Preoperative assessment of liver function: a comparison of 99mTc-Mebrofenin scintigraphy with indocyanine green clearance test. *Liver Int* 2004;24(2):117-23. doi: 10.1111/j.1478-3231.2004.0901.x [published Online First: 2004/04/14]
11. Fernandez FG, Ritter J, Goodwin JW, et al. Effect of steatohepatitis associated with irinotecan or oxaliplatin pretreatment on resectability of hepatic colorectal metastases. *J Am Coll Surg* 2005;200(6):845-53. doi: 10.1016/j.jamcollsurg.2005.01.024 [published Online First: 2005/06/01]
12. Schindl MJ, Redhead DN, Fearon KC, et al. The value of residual liver volume as a predictor of hepatic dysfunction and infection after major liver resection. *Gut* 2005;54(2):289-96. doi: 10.1136/gut.2004.046524 [published Online First: 2005/01/14]
13. Yigitler C, Farges O, Kianmanesh R, et al. The small remnant liver after major liver resection: how common and how relevant? *Liver Transpl* 2003;9(9):S18-25. doi: 10.1053/jlts.2003.50194 [published Online First: 2003/08/28]
14. Farges O, Belghiti J, Kianmanesh R, et al. Portal vein embolization before right hepatectomy: prospective clinical trial. *Ann Surg* 2003;237(2):208-17. doi: 10.1097/01.SLA.0000048447.16651.7B [published Online First: 2003/02/01]
15. Wakabayashi H, Ishimura K, Okano K, et al. Application of preoperative portal vein embolization before major hepatic resection in patients with normal or abnormal liver parenchyma. *Surgery* 2002;131(1):26-33. [published Online First: 2002/01/29]

For peer review only

16. Folprecht G, Gruenberger T, Bechstein WO, et al. Tumour response and secondary resectability of colorectal liver metastases following neoadjuvant chemotherapy with cetuximab: the CELIM randomised phase 2 trial. *Lancet Oncol* 2010;11(1):38-47. doi: 10.1016/S1470-2045(09)70330-4 [published Online First: 2009/11/28]
17. Fausto N, Campbell JS, Riehle KJ. Liver regeneration. *Hepatology* 2006;43(2 Suppl 1):S45-53. doi: 10.1002/hep.20969 [published Online First: 2006/02/01]
18. Ikeda Y, Matsumata T, Takenaka K, et al. Preliminary report of tumor metastasis during liver regeneration after hepatic resection in rats. *Eur J Surg Oncol* 1995;21(2):188-90. [published Online First: 1995/04/01]
19. Nagashima I, Takada T, Adachi M, et al. Proposal of criteria to select candidates with colorectal liver metastases for hepatic resection: comparison of our scoring system to the positive number of risk factors. *World J Gastroenterol* 2006;12(39):6305-9. [published Online First: 2006/10/31]
20. Schneider PD. Preoperative assessment of liver function. *Surg Clin North Am* 2004;84(2):355-73. doi: 10.1016/S0039-6109(03)00224-X [published Online First: 2004/04/06]
21. Khan AZ, Toogood GJ. The case for adjuvant chemotherapy. Against. *Ann R Coll Surg Engl* 2008;90(6):454-5. [published Online First: 2008/09/09]
22. Morris EJ, Forman D, Thomas JD, et al. Surgical management and outcomes of colorectal cancer liver metastases. *Br J Surg* 2010;97(7):1110-8. doi: 10.1002/bjs.7032 [published Online First: 2010/07/16]
23. Mattar RE, Al-Alem F, Simoneau E, et al. Preoperative selection of patients with colorectal cancer liver metastasis for hepatic resection. *World J Gastroenterol* 2016;22(2):567-81. doi: 10.3748/wjg.v22.i2.567 [published Online First: 2016/01/27]
24. Abdalla EK, Vauthey JN, Ellis LM, et al. Recurrence and outcomes following hepatic resection, radiofrequency ablation, and combined resection/ablation for colorectal liver metastases. *Ann Surg* 2004;239(6):818-25; discussion 25-7. [published Online First: 2004/05/29]
25. Choti MA, Sitzmann JV, Tiburi MF, et al. Trends in long-term survival following liver resection for hepatic colorectal metastases. *Ann Surg* 2002;235(6):759-66. [published Online First: 2002/05/30]
26. Fernandez FG, Drebin JA, Linehan DC, et al. Five-year survival after resection of hepatic metastases from colorectal cancer in patients screened by positron emission tomography with F-18 fluorodeoxyglucose (FDG-PET). *Ann Surg* 2004;240(3):438-47; discussion 47-50. [published Online First: 2004/08/21]
27. Pawlik TM, Scoggins CR, Zorzi D, et al. Effect of surgical margin status on survival and site of recurrence after hepatic resection for colorectal metastases. *Ann Surg* 2005;241(5):715-22, discussion 22-4. [published Online First: 2005/04/26]
28. Simmonds PC, Primrose JN, Colquitt JL, et al. Surgical resection of hepatic metastases from colorectal cancer: a systematic review of published studies. *Br J Cancer* 2006;94(7):982-99. doi: 10.1038/sj.bjc.6603033 [published Online First: 2006/03/16]
29. Nordlinger B, Van Cutsem E, Rougier P, et al. Does chemotherapy prior to liver resection increase the potential for cure in patients with metastatic colorectal cancer? A report from the European Colorectal Metastases Treatment Group. *Eur J Cancer* 2007;43(14):2037-45. doi: 10.1016/j.ejca.2007.07.017 [published Online First: 2007/09/04]
30. Adam R, Delvart V, Pascal G, et al. Rescue surgery for unresectable colorectal liver metastases downstaged by chemotherapy: a model to predict long-term survival. *Ann Surg* 2004;240(4):644-57; discussion 57-8. [published Online First: 2004/09/24]
31. Adam R, Pascal G, Castaing D, et al. Tumor progression while on chemotherapy: a contraindication to liver resection for multiple colorectal metastases? *Ann Surg* 2004;240(6):1052-61; discussion 61-4. [published Online First: 2004/12/01]
32. Abdalla EK, Barnett CC, Doherty D, et al. Extended hepatectomy in patients with hepatobiliary malignancies with and without preoperative portal vein embolization. *Arch Surg* 2002;137(6):675-80; discussion 80-1. [published Online First: 2002/06/07]

For peer review only - http://bmjopen.bmj.com/site/about/guidelines.xhtml

33. Jaeck D, Oussoultzoglou E, Rosso E, et al. A two-stage hepatectomy procedure combined with portal vein embolization to achieve curative resection for initially unresectable multiple and bilobar colorectal liver metastases. *Ann Surg* 2004;240(6):1037-49; discussion 49-51. [published Online First: 2004/12/01]
34. Adam R, Avisar E, Ariche A, et al. Five-year survival following hepatic resection after neoadjuvant therapy for nonresectable colorectal. *Ann Surg Oncol* 2001;8(4):347-53. [published Online First: 2001/05/16]
35. Masi G, Cupini S, Marcucci L, et al. Treatment with 5-fluorouracil/folinic acid, oxaliplatin, and irinotecan enables surgical resection of metastases in patients with initially unresectable metastatic colorectal cancer. *Ann Surg Oncol* 2006;13(1):58-65. doi: 10.1245/ASO.2006.03.094 [published Online First: 2005/12/24]
36. Nordlinger B, Sorbye H, Glimelius B, et al. Perioperative chemotherapy with FOLFOX4 and surgery versus surgery alone for resectable liver metastases from colorectal cancer (EORTC Intergroup trial 40983): a randomised controlled trial. *Lancet* 2008;371(9617):1007-16. doi: 10.1016/S0140-6736(08)60455-9 [published Online First: 2008/03/25]
37. Zeiss J, Merrick HW, Savolaine ER, et al. Fatty liver change as a result of hepatic artery infusion chemotherapy. *Am J Clin Oncol* 1990;13(2):156-60. [published Online First: 1990/04/01]
38. Moertel CG, Fleming TR, Macdonald JS, et al. Hepatic toxicity associated with fluorouracil plus levamisole adjuvant therapy. *J Clin Oncol* 1993;11(12):2386-90. doi: 10.1200/JCO.1993.11.12.2386 [published Online First: 1993/12/01]
39. Sorensen P, Edal AL, Madsen EL, et al. Reversible hepatic steatosis in patients treated with interferon alfa-2a and 5-fluorouracil. *Cancer* 1995;75(10):2592-6. [published Online First: 1995/05/15]
40. Peppercorn PD, Reznek RH, Wilson P, et al. Demonstration of hepatic steatosis by computerized tomography in patients receiving 5-fluorouracil-based therapy for advanced colorectal cancer. *Br J Cancer* 1998;77(11):2008-11. [published Online First: 1998/07/17]
41. Behrns KE, Tsiotos GG, DeSouza NF, et al. Hepatic steatosis as a potential risk factor for major hepatic resection. *J Gastrointest Surg* 1998;2(3):292-8. [published Online First: 1998/12/08]
42. Belghiti J, Hiramatsu K, Benoist S, et al. Seven hundred forty-seven hepatectomies in the 1990s: an update to evaluate the actual risk of liver resection. *J Am Coll Surg* 2000;191(1):38-46. [published Online First: 2000/07/18]
43. Parikh AA, Gentner B, Wu TT, et al. Perioperative complications in patients undergoing major liver resection with or without neoadjuvant chemotherapy. *J Gastrointest Surg* 2003;7(8):1082-8. [published Online First: 2003/12/17]
44. Kooby DA, Fong Y, Suriawinata A, et al. Impact of steatosis on perioperative outcome following hepatic resection. *J Gastrointest Surg* 2003;7(8):1034-44. [published Online First: 2003/12/17]
45. Rubbia-Brandt L, Audard V, Sartoretti P, et al. Severe hepatic sinusoidal obstruction associated with oxaliplatin-based chemotherapy in patients with metastatic colorectal cancer. *Ann Oncol* 2004;15(3):460-6. [published Online First: 2004/03/05]
46. Karoui M, Penna C, Amin-Hashem M, et al. Influence of preoperative chemotherapy on the risk of major hepatectomy for colorectal liver metastases. *Ann Surg* 2006;243(1):1-7. [published Online First: 2005/12/24]
47. Vauthey JN, Pawlik TM, Ribero D, et al. Chemotherapy regimen predicts steatohepatitis and an increase in 90-day mortality after surgery for hepatic colorectal metastases. *J Clin Oncol* 2006;24(13):2065-72. doi: 10.1200/jco.2005.05.3074 [published Online First: 2006/05/02]
48. Pawlik TM, Olino K, Gleisner AL, et al. Preoperative chemotherapy for colorectal liver metastases: impact on hepatic histology and postoperative outcome. *J Gastrointest Surg* 2007;11(7):860-8. doi: 10.1007/s11605-007-0149-4 [published Online First: 2007/05/12]

For peer review only

49. Hewes JC, Dighe S, Morris RW, et al. Preoperative chemotherapy and the outcome of liver resection for colorectal metastases. *World J Surg* 2007;31(2):353-64; discussion 65-6. doi: 10.1007/s00268-006-0103-8 [published Online First: 2007/01/16]
50. Nakano H, Oussoultzoglou E, Rosso E, et al. Sinusoidal injury increases morbidity after major hepatectomy in patients with colorectal liver metastases receiving preoperative chemotherapy. *Ann Surg* 2008;247(1):118-24. doi: 10.1097/SLA.0b013e31815774de [published Online First: 2007/12/25]
51. Hewes J. Re: Preoperative chemotherapy and the outcome of liver resection for colorectal metastases. *World J Surg* 2007;31(9):1902; author reply 03. doi: 10.1007/s00268-007-9065-8 [published Online First: 2007/05/10]
52. Soubrane O, Brouquet A, Zalinski S, et al. Predicting high grade lesions of sinusoidal obstruction syndrome related to oxaliplatin-based chemotherapy for colorectal liver metastases: correlation with post-hepatectomy outcome. *Ann Surg* 2010;251(3):454-60. doi: 10.1097/SLA.0b013e3181c79403 [published Online First: 2010/02/18]
53. Tamandl D, Klinger M, Eipeldauer S, et al. Sinusoidal obstruction syndrome impairs long-term outcome of colorectal liver metastases treated with resection after neoadjuvant chemotherapy. *Ann Surg Oncol* 2011;18(2):421-30. doi: 10.1245/s10434-010-1317-4 [published Online First: 2010/09/17]
54. Wolf PS, Park JO, Bao F, et al. Preoperative chemotherapy and the risk of hepatotoxicity and morbidity after liver resection for metastatic colorectal cancer: a single institution experience. *J Am Coll Surg* 2013;216(1):41-9. doi: 10.1016/j.jamcollsurg.2012.08.030 [published Online First: 2012/10/09]
55. Parkin E, O'Reilly DA, Adam R, et al. Equivalent survival in patients with and without steatosis undergoing resection for colorectal liver metastases following pre-operative chemotherapy. *Eur J Surg Oncol* 2014 doi: 10.1016/j.ejso.2014.07.040 [published Online First: 2014/09/06]
56. Vigano L, Capussotti L, De Rosa G, et al. Liver resection for colorectal metastases after chemotherapy: impact of chemotherapy-related liver injuries, pathological tumor response, and micrometastases on long-term survival. *Ann Surg* 2013;258(5):731-40; discussion 41-2. doi: 10.1097/SLA.0b013e3182a6183e [published Online First: 2013/09/21]
57. Vigano L, De Rosa G, Toso C, et al. Reversibility of chemotherapy-related liver injury. *J Hepatol* 2017;67(1):84-91. doi: 10.1016/j.jhep.2017.02.031 [published Online First: 2017/03/13]
58. Welsh FK, Tilney HS, Tekkis PP, et al. Safe liver resection following chemotherapy for colorectal metastases is a matter of timing. *Br J Cancer* 2007;96(7):1037-42. doi: 10.1038/sj.bjc.6603670 [published Online First: 2007/03/14]
59. Poon RT, Fan ST, Lo CM, et al. Extended hepatic resection for hepatocellular carcinoma in patients with cirrhosis: is it justified? *Ann Surg* 2002;236(5):602-11. doi: 10.1097/01.SLA.0000033038.38956.5E [published Online First: 2002/11/01]
60. Farges O, Malassagne B, Flejou JF, et al. Risk of major liver resection in patients with underlying chronic liver disease: a reappraisal. *Ann Surg* 1999;229(2):210-5. [published Online First: 1999/02/19]
61. van den Broek MA, Olde Damink SW, Dejong CH, et al. Liver failure after partial hepatic resection: definition, pathophysiology, risk factors and treatment. *Liver Int* 2008;28(6):767-80. doi: 10.1111/j.1478-3231.2008.01777.x [published Online First: 2008/07/24]
62. Ryan P, Nanji S, Pollett A, et al. Chemotherapy-induced liver injury in metastatic colorectal cancer: semiquantitative histologic analysis of 334 resected liver specimens shows that vascular injury but not steatohepatitis is associated with preoperative chemotherapy. *Am J Surg Pathol* 2010;34(6):784-91. doi: 10.1097/PAS.0b013e3181dc242c [published Online First: 2010/04/28]
63. Stockmann M, Lock JF, Malinowski M, et al. The LiMAx test: a new liver function test for predicting postoperative outcome in liver surgery. *HPB (Oxford)* 2010;12(2):139-46. doi: 10.1111/j.1477-2574.2009.00151.x [published Online First: 2010/05/25]

For peer review only

64. Jiang Z, Dragin N, Jorge-Nebert LF, et al. Search for an association between the human CYP1A2 genotype and CYP1A2 metabolic phenotype. *Pharmacogenet Genomics* 2006;16(5):359-67. doi: 10.1097/01.fpc.0000204994.99429.46 [published Online First: 2006/04/13]
65. Palmer CN, Coates PJ, Davies SE, et al. Localization of cytochrome P-450 gene expression in normal and diseased human liver by in situ hybridization of wax-embedded archival material. *Hepatology* 1992;16(3):682-7. [published Online First: 1992/09/01]
66. George J, Murray M, Byth K, et al. Differential alterations of cytochrome P450 proteins in livers from patients with severe chronic liver disease. *Hepatology* 1995;21(1):120-8. [published Online First: 1995/01/01]
67. Schoeller DA, Schneider JF, Solomons NW, et al. Clinical diagnosis with the stable isotope 13C in CO2 breath tests: methodology and fundamental considerations. *J Lab Clin Med* 1977;90(3):412-21. [published Online First: 1977/09/01]
68. Stockmann M, Lock JF, Riecke B, et al. Prediction of postoperative outcome after hepatectomy with a new bedside test for maximal liver function capacity. *Ann Surg* 2009;250(1):119-25. doi: 10.1097/SLA.0b013e3181ad85b5 [published Online First: 2009/06/30]
69. Lock JF, Malinowski M, Seehofer D, et al. Function and volume recovery after partial hepatectomy: influence of preoperative liver function, residual liver volume, and obesity. *Langenbecks Arch Surg* 2012;397(8):1297-304. doi: 10.1007/s00423-012-0972-2 [published Online First: 2012/06/26]
70. Jara M, Reese T, Malinowski M, et al. Reductions in post-hepatectomy liver failure and related mortality after implementation of the LiMAx algorithm in preoperative work-up: a single-centre analysis of 1170 hepatectomies of one or more segments. *HPB (Oxford)* 2015;17(7):651-8. doi: 10.1111/hpb.12424 [published Online First: 2015/06/11]
71. Jara M, Bednarsch J, Valle E, et al. Reliable assessment of liver function using LiMAx. *J Surg Res* 2015;193(1):184-9. doi: 10.1016/j.jss.2014.07.041 [published Online First: 2014/08/26]
72. Jara M, Bednarsch J, Malinowski M, et al. Effects of oxaliplatin-based chemotherapy on liver function--an analysis of impact and functional recovery using the LiMAx test. *Langenbecks Arch Surg* 2016;401(1):33-41. doi: 10.1007/s00423-015-1352-5 [published Online First: 2015/10/29]
73. Lock JF, Westphal T, Rubin T, et al. LiMAx Test Improves Diagnosis of Chemotherapy-Associated Liver Injury Before Resection of Colorectal Liver Metastases. *Ann Surg Oncol* 2017;24(9):2447-55. doi: 10.1245/s10434-017-5887-2 [published Online First: 2017/05/19]
74. Foster KJ, Dewbury KC, Griffith AH, et al. The accuracy of ultrasound in the detection of fatty infiltration of the liver. *Br J Radiol* 1980;53(629):440-2. doi: 10.1259/0007-1285-53-629-440 [published Online First: 1980/05/01]
75. Bydder GM, Kreel L, Chapman RW, et al. Accuracy of computed tomography in diagnosis of fatty liver. *Br Med J* 1980;281(6247):1042. [published Online First: 1980/10/18]
76. Piekarski J, Goldberg HI, Royal SA, et al. Difference between liver and spleen CT numbers in the normal adult: its usefulness in predicting the presence of diffuse liver disease. *Radiology* 1980;137(3):727-9. doi: 10.1148/radiology.137.3.6934563 [published Online First: 1980/12/01]
77. Meek DR, Mills PR, Gray HW, et al. A comparison of computed tomography, ultrasound and scintigraphy in the diagnosis of alcoholic liver disease. *Br J Radiol* 1984;57(673):23-7. doi: 10.1259/0007-1285-57-673-23 [published Online First: 1984/01/01]
78. Graif M, Yanuka M, Baraz M, et al. Quantitative estimation of attenuation in ultrasound video images: correlation with histology in diffuse liver disease. *Invest Radiol* 2000;35(5):319-24. [published Online First: 2000/05/10]

For peer review only

79. Reeder SB, Cruite I, Hamilton G, et al. Quantitative Assessment of Liver Fat with Magnetic Resonance Imaging and Spectroscopy. *J Magn Reson Imaging* 2011;34(4):spcone. doi: 10.1002/jmri.22775 [published Online First: 2011/10/26]
80. Davies RJ, Saverymuttu SH, Fallowfield M, et al. Paradoxical lack of ultrasound attenuation with gross fatty change in the liver. *Clin Radiol* 1991;43(6):393-6. [published Online First: 1991/06/01]
81. Thomsen C, Becker U, Winkler K, et al. Quantification of liver fat using magnetic resonance spectroscopy. *Magn Reson Imaging* 1994;12(3):487-95. [published Online First: 1994/01/01]
82. Hatfield MK, Beres RA, Sane SS, et al. Percutaneous imaging-guided solid organ core needle biopsy: coaxial versus noncoaxial method. *AJR Am J Roentgenol* 2008;190(2):413-7. doi: 10.2214/AJR.07.2676 [published Online First: 2008/01/24]
83. Ratziu V, Charlotte F, Heurtier A, et al. Sampling variability of liver biopsy in nonalcoholic fatty liver disease. *Gastroenterology* 2005;128(7):1898-906. [published Online First: 2005/06/09]
84. El-Badry AM, Breitenstein S, Jochum W, et al. Assessment of hepatic steatosis by expert pathologists: the end of a gold standard. *Ann Surg* 2009;250(5):691-7. doi: 10.1097/SLA.0b013e3181bcd6dd [published Online First: 2009/10/07]
85. Bravo AA, Sheth SG, Chopra S. Liver biopsy. *N Engl J Med* 2001;344(7):495-500. doi: 10.1056/NEJM200102153440706 [published Online First: 2001/02/15]
86. Haaga JR. Magnetic resonance imaging of the liver. *Radiol Clin North Am* 1984;22(4):879-90. [published Online First: 1984/12/01]
87. Stark DD, Moss AA, Goldberg HI. Nuclear magnetic resonance of the liver, spleen, and pancreas. *Cardiovasc Intervent Radiol* 1986;8(5-6):329-41. [published Online First: 1986/01/01]
88. Kanzer GK, Weinreb JC. Magnetic resonance imaging of diseases of the liver and biliary system. *Radiol Clin North Am* 1991;29(6):1259-84. [published Online First: 1991/11/01]
89. Lee JK, Dixon WT, Ling D, et al. Fatty infiltration of the liver: demonstration by proton spectroscopic imaging. Preliminary observations. *Radiology* 1984;153(1):195-201. doi: 10.1148/radiology.153.1.6089264 [published Online First: 1984/10/01]
90. Kukuk GM, Hittatiya K, Sprinkart AM, et al. Comparison between modified Dixon MRI techniques, MR spectroscopic relaxometry, and different histologic quantification methods in the assessment of hepatic steatosis. *Eur Radiol* 2015;25(10):2869-79. doi: 10.1007/s00330-015-3703-6 [published Online First: 2015/04/24]
91. Szczepaniak LS, Nurenberg P, Leonard D, et al. Magnetic resonance spectroscopy to measure hepatic triglyceride content: prevalence of hepatic steatosis in the general population. *Am J Physiol Endocrinol Metab* 2005;288(2):E462-8. doi: 10.1152/ajpendo.00064.2004 [published Online First: 2004/09/02]
92. Clark JM, Brancati FL, Diehl AM. Nonalcoholic fatty liver disease. *Gastroenterology* 2002;122(6):1649-57. [published Online First: 2002/05/23]
93. Szczepaniak LS, Babcock EE, Schick F, et al. Measurement of intracellular triglyceride stores by H spectroscopy: validation in vivo. *Am J Physiol* 1999;276(5 Pt 1):E977-89. [published Online First: 1999/05/18]
94. Bohte AE, van Werven JR, Bipat S, et al. The diagnostic accuracy of US, CT, MRI and 1H-MRS for the evaluation of hepatic steatosis compared with liver biopsy: a meta-analysis. *Eur Radiol* 2011;21(1):87-97. doi: 10.1007/s00330-010-1905-5 [published Online First: 2010/08/04]
95. Banerjee R, Pavlides M, Tunnicliffe EM, et al. Multiparametric magnetic resonance for the non-invasive diagnosis of liver disease. *J Hepatol* 2014;60(1):69-77. doi: 10.1016/j.jhep.2013.09.002 [published Online First: 2013/09/17]
96. McPherson S, Jonsson JR, Cowin GJ, et al. Magnetic resonance imaging and spectroscopy accurately estimate the severity of steatosis provided the stage of fibrosis is considered. *J Hepatol* 2009;51(2):389-97. doi: 10.1016/j.jhep.2009.04.012 [published Online First: 2009/06/10]

For peer review only

97. Valls C, Iannacconne R, Alba E, et al. Fat in the liver: diagnosis and characterization. *Eur Radiol* 2006;16(10):2292-308. doi: 10.1007/s00330-006-0146-0 [published Online First: 2006/02/16]
98. van Werven JR, Hoogduin JM, Nederveen AJ, et al. Reproducibility of 3.0 Tesla magnetic resonance spectroscopy for measuring hepatic fat content. *J Magn Reson Imaging* 2009;30(2):444-8. doi: 10.1002/jmri.21837 [published Online First: 2009/07/25]
99. Longo R, Pollesello P, Ricci C, et al. Proton MR spectroscopy in quantitative in vivo determination of fat content in human liver steatosis. *J Magn Reson Imaging* 1995;5(3):281-5. [published Online First: 1995/05/01]
100. Longo R, Ricci C, Masutti F, et al. Fatty infiltration of the liver. Quantification by 1H localized magnetic resonance spectroscopy and comparison with computed tomography. *Invest Radiol* 1993;28(4):297-302. [published Online First: 1993/04/01]
101. Idilman IS, Aniktar H, Idilman R, et al. Hepatic steatosis: quantification by proton density fat fraction with MR imaging versus liver biopsy. *Radiology* 2013;267(3):767-75. doi: 10.1148/radiol.13121360 [published Online First: 2013/02/06]
102. Tang A, Tan J, Sun M, et al. Nonalcoholic fatty liver disease: MR imaging of liver proton density fat fraction to assess hepatic steatosis. *Radiology* 2013;267(2):422-31. doi: 10.1148/radiol.12120896 [published Online First: 2013/02/06]
103. Noureddin M, Lam J, Peterson MR, et al. Utility of magnetic resonance imaging versus histology for quantifying changes in liver fat in nonalcoholic fatty liver disease trials. *Hepatology* 2013;58(6):1930-40. doi: 10.1002/hep.26455 [published Online First: 2013/05/23]
104. Parkin E, O'Reilly DA, Plumb AA, et al. Digital histology quantification of intra-hepatic fat in patients undergoing liver resection. *Eur J Surg Oncol* 2015;41(8):1020-7. doi: 10.1016/j.ejso.2015.05.003 [published Online First: 2015/06/10]
105. Coe P, Williams S, Morris D, et al. Development of MR quantified pancreatic fat deposition as a cancer risk biomarker (PanORAMA) [submitted]. *Pancreatology* 2018

For peer review only

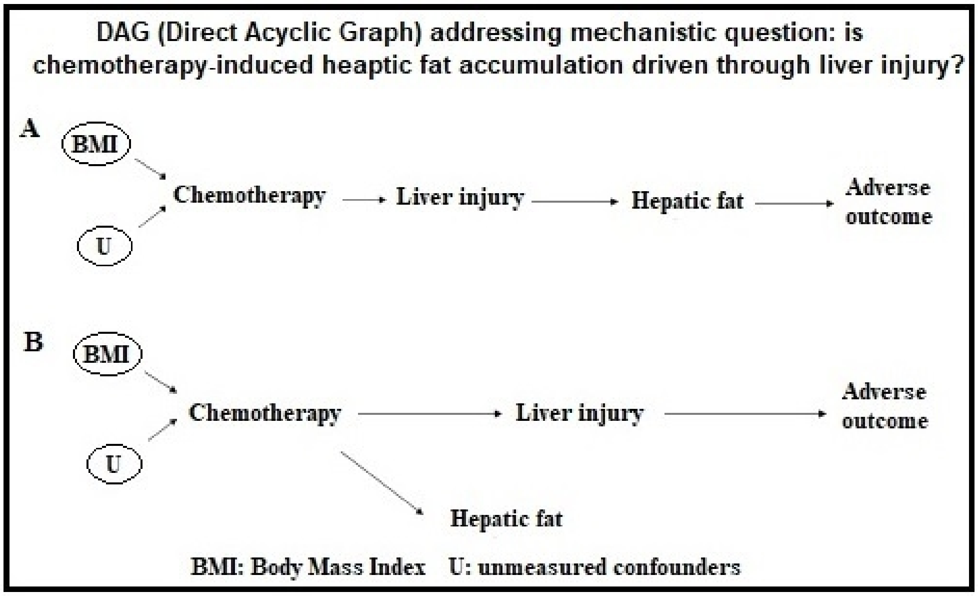

Direct Acyclic Graph

43x27mm (300 x 300 DPI)

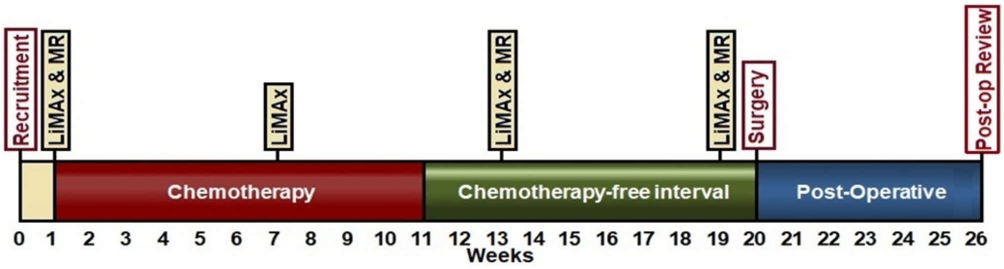

Study Participant Timeline

87x23mm (300 x 300 DPI)

APPENDIX 1 - STUDY SCHEDULE

			Standard care assessments			Experimental added assessments		
**Timeline (weeks)**		**Oncology Outpatient Department**	**Blood Tests**	**CT**	**Histology**	**LiMAx**	**MR**	**EQ-5D**
**BASELINE**		**Patient identification HPB MDT/ Oncology**		✓				
		**New Patient Oncology Clinic – CLiFF Study information**	✓					
**1**	Day 1	Oncology Clinic – Assessment, Bloods	✓			✓	✓	✓
	Day 2	**Chemotherapy- Cycle 1**						
**3**	Day 1	Oncology Clinic – Assessment, Bloods	✓					
	Day 2	**Chemotherapy- Cycle 2**						
**5**	Day 1	Oncology Clinic – Assessment, Bloods	✓					
	Day 2	**Chemotherapy- Cycle 3**						
**7**	Day 1	Oncology Clinic – Assessment, Bloods	✓			✓		
	Day 2	**Chemotherapy- Cycle 4**						
**9**	Day 1	Oncology Clinic – Assessment, Bloods	✓					
	Day 2	**Chemotherapy- Cycle 5**						
**11**	Day 1	Oncology Clinic – Assessment, Bloods	✓					
	Day 2	**Chemotherapy- Cycle 6**						
**13**	*Chemo finished	Oncology Clinic – Final Assessment, Bloods **<u>ONCOLOGY DISCHARGE</u>**	✓	✓		✓	✓	✓
**19**	**Post-Chemotherapy Week 8**							
	**Additional Visit to Christie Site** LiMAx and MR					✓	✓	✓
**Pre-Op** (~ Wk 19/20)		Pre-operative assessment clinic at Manchester Foundation Trust HPB Dept	✓					
**6 weeks post-op** (~ Wk 26)		Post-operative review clinic at Manchester Foundation Trust HPB Dept	✓		✓			✓

APPENDIX 2 – STUDY ASSESSMENTS

	Event	Baseline	Pre-chemo	2nd cycle	3rd cycle	4th cycle	5th cycle	6th cycle	Following last cycle of chemotherapy	Approx 8 wks post-chemo/ pre-op assessment	Post-op Assessment
**Standard Care Events/ Assessments**	History	✓									
	Physical examination	✓	✓	✓	✓	✓	✓	✓	✓	✓	✓
	Height	✓									
	Weight	✓	✓	✓	✓	✓	✓	✓	✓	✓	✓
	WHO perf score	✓	✓	✓	✓	✓	✓	✓	✓	✓	✓
	FBC	✓	✓	✓	✓	✓	✓	✓	✓	✓	✓
	Biochemistry [b]	✓	✓	✓	✓	✓	✓	✓	✓	✓	✓
	CEA	✓								✓	
	Coagulation [c]	✓	✓	✓	✓	✓	✓	✓	✓	✓	
	Pregnancy test [d]	✓									
	CT	✓							✓		
	Histopathology										✓
**Experimental Additional Events/ Assessments**	Informed Consent		✓								
	EQ-5D Score		✓						✓	✓	✓
	MR		✓						✓	✓	
	LiMAx		✓			✓			✓	✓	

For peer review only - http://bmjopen.bmj.com/site/about/guidelines.xhtml

BMJ Open

A prospective study of Change in Liver Function and Fat in patients with colorectal liver metastases undergoing pre-operative chemotherapy: protocol for the CLiFF study

Journal:	*BMJ Open*
Manuscript ID	bmjopen-2018-027630.R1
Article Type:	Protocol
Date Submitted by the Author:	08-Apr-2019
Complete List of Authors:	Parmar, Kat; University of Manchester, Division of Cancer Sciences, School of Medical Sciences, Faculty of Biology, Medicine and Health; Manchester Cancer Research Centre<br>O'Reilly, Derek; Central Manchester University Hospitals NHS Foundation Trust, Hepatobiliary Surgery<br>Valle, Juan; Christie NHS Foundation Trust, Oncology; University of Manchester, Division of Cancer Sciences, School of Medical Sciences, Faculty of Biology, Medicine and Health<br>Braun, Michael; Christie NHS Foundation Trust, Oncology<br>Naish, Jo; University of Manchester Institute of Cardiovascular Sciences<br>Williams, Steve; University of Manchester Centre for Imaging Sciences<br>Lloyd, William; University of Manchester Centre for Imaging Sciences<br>Malcomson, Lee; University of Manchester, Division of Cancer Sciences, School of Medical Sciences, Faculty of Biology, Medicine and Health; Christie NHS Foundation Trust<br>Cresswell, Katharine; Manchester University NHS Foundation Trust, Public Programmes Team, Research and Innovation Division<br>Bamford, Colin; NIHR Manchester Biomedical Research Centre, Cancer Patient and Public Advisory Group<br>Renehan, Andrew; University of Manchester, 1 Division of Cancer Sciences, School of Medical Sciences, Faculty of Biology, Medicine and Health; Christie NHS Foundation Trust
**Primary Subject Heading**:	Surgery
Secondary Subject Heading:	Surgery, Oncology, Radiology and imaging, Diagnostics
Keywords:	Colorectal surgery < SURGERY, Hepatobiliary surgery < SURGERY, CHEMOTHERAPY

SCHOLARONE™
Manuscripts

Type of article: study protocol (formatted for BMJ Open)

A prospective study of Change in Liver Function and Fat in patients with colorectal liver metastases undergoing pre-operative chemotherapy: protocol for the CLiFF study

Kat L Parmar,[1,2,3,4] Derek O'Reilly,[4] Juan W Valle,[1,3] Michael Braun,[3] Jo H Naish,[5] Steve R Williams,[6] William K Lloyd,[6] Lee Malcomson,[1,2,3] Katharine Cresswell,[2,7,8] Colin Bamford,[9] Andrew G Renehan[1,2,3]

[1] Division of Cancer Sciences, School of Medical Sciences, Faculty of Biology, Medicine and Health, University of Manchester

[2] Manchester Cancer Research Centre, NIHR Manchester Biomedical Research Centre, Manchester

[3] The Christie NHS Foundation Trust, Manchester

[4] Department of Hepatobiliary Surgery, Manchester University Foundation Hospitals, Manchester

[5] Division of Cardiovascular Sciences, School of Medical Science, Faculty of Biology, Medicine and Health, University of Manchester

[6] Centre for Imaging Sciences, School of Health Sciences, Faculty of Biology, Medicine and Health, University of Manchester

[7] Division of Evolution and Genomic Sciences, School of Biological Sciences, Faculty of Biology, Medicine and Health, University of Manchester

[8] Public Programmes Team, Research and Innovation Division, Manchester University NHS Foundation Trust, Manchester

[9] NIHR Manchester Biomedical Research Centre Cancer Patient and Public Advisory Group

Running title: *Liver fat and function after chemotherapy*

Keywords: liver injury, liver steatosis, liver resection, colorectal cancer metastases, chemotherapy

Correspondence to:

Miss Kat Parmar
Division of Cancer Sciences, School of Medical Sciences,
Faculty of Biology, Medicine and Health,
University of Manchester,
The Christie NHS Foundation Trust,
Wilmslow Road
Manchester,
M20 4BX UK
Tel: +44 161 3060870
E-mail: kat.parmar@manchester.ac.uk

Abstract: 266 words (max: 300); main text: 3818 words (max: 4000); x2 figures; 105 references supplemental material (2 pages); language: UK English.

For peer review only

ABSTRACT

Introduction

Pre-operative chemotherapy in patients undergoing resection for colorectal liver metastases (CLM) improves oncological outcomes. However, chemotherapy-associated liver injury (occurring in two patterns: vascular and fat deposition) is a real clinical concern prior to hepatic resection. After major liver resection, regeneration of the residual liver is a prerequisite for recovery and avoidance of liver failure, but this regenerative capacity may be hindered by chemotherapy. Thus, there is a need to predict for this serious complication. Over the past two decades, several tests and derived indices have been developed, which have failed to achieve clinical utility, mainly as they were indirect measurements of liver function. Here, we will use a novel test of liver function (the LiMAx test), and measure liver fat using magnetic resonance (MR) imaging.

Methods and analysis

This prospective study will assess changes in liver function longitudinally, measured by the LiMAx test, and liver fat, measured by advanced MR imaging using both MR spectroscopy and the modified Dixon method, in up to 35 patients undergoing pre-operative chemotherapy for CLM. The primary outcomes will be the changes in liver function and fat compared to baseline pre-chemotherapy measurements. Secondary outcome measures include: routinely measured liver function blood tests, anthropometric measurements, post-operative histology and digital quantification of fat, post-operative complications and mortality and quality of life.

Ethics and dissemination

The study was approved by a National Health Service Research Ethics Committee and registered with the Health Research Authority. Dissemination will be via international and national conferences, and the NIHR network. Manuscripts will be published.

Trial Registration Number:

This study is registered online at www.clinicaltrials.gov (registration number NCT03562234).

For peer review only - http://bmjopen.bmj.com/site/about/guidelines.xhtml

Strengths and limitations of this study

- This is an evaluation of the effects of pre-operative chemotherapy upon liver function and fat using novel techniques, addressing the important clinical issue of liver injury prior to hepatic resection
- These non-invasive techniques permit repeated measurements to assess change in liver function and fat over time, permitting interpretation of causality – this has not been possible in the past
- Chemotherapy-related changes in liver fat and function will be assessed for reversibility and correlated with both standard assessment measures and post-operative histology, providing insight into the scale of the injuries prior to the post-chemotherapy recovery period
- Changes and recovery in liver fat and function will be correlated with post-operative morbidity and mortality
- A potential limitation is that this is a single-site observational study with a relatively small sample size

For peer review only - http://bmjopen.bmj.com/site/about/guidelines.xhtml

Background

Colorectal cancer (CRC) is the second most common cause of cancer death in the UK.[1] Death is usually due to metastatic disease, with the most common site being the liver (colorectal liver metastases - CLM). There is an incidence of 15-20% CLM at initial diagnosis of CRC[2] with up to 50% of patients ultimately developing CLM.[3-5 6]

Surgical resection of CLM is the only single-modality therapy associated with cure and remains the mainstay of treatment.[7] The resectability of CLM depends upon the volume and function of the future liver remnant (FLR). Volume alone is not an ideal assessment of liver functionality as it cannot reliably predict outcome.[7-11 12] FLR function is decreased in patients with parenchymal disease, despite being of equal volume to patients with heathy livers,[13-15] and liver disease may result in impaired liver regeneration.[7 11 16-18] In the presence of impaired liver function the surgical strategy may need to be adapted, either to increase the volume of the FLR or perform a less extensive resection.[2 19] Accurate pre-operative assessment of liver function is therefore crucial.[20]

With best supportive care, the median survival of patients with CLMs is 6-13 months (where five-year survival is rare).[21] In patients fit enough to receive combined hepatic resection and chemotherapy, the 5-year survival improves to 50%.[22 23 24 24-27] Pre-operative chemotherapy is used in the setting of both resectable and unresectable CLM, with the intentions of (i) improving survival (neoadjuvant approach) in operable CLM; and (ii) converting inoperable CLM into operable (downsizing approach). Approximately 70% of patients presenting with CLM are initially deemed unresectable,[28] and after systemic chemotherapy, up to 40% of patients may be judged resectable.[61 29 30-33] Outcomes in these “downsized” cases can be similar to those initially presenting with resectable disease.[34 35] In the setting of operable CLM, the randomized EPOC trial reported a 7.3% improvement in 3-year progression-free survival with addition of chemotherapy versus resection alone for CLM, and highlighted the

For peer review only - http://bmjopen.bmj.com/site/about/guidelines.xhtml

issue that organ-specific resectional surgery does not address the systemic process, leading to high post-operative recurrence rates.[36]

The main concern regarding the pre-operative administration of chemotherapy for CLM is direct pathologic changes to the liver, known as chemotherapy-associated liver injury (CALI). CALIs comprise two main types of liver injury: vascular changes and fatty changes. These injuries are not mutually exclusive; patients who have developed fatty changes can simultaneously develop vascular changes.[7] There are many inconsistent reports on the types of liver injury seen and their associations with specific chemotherapy regimens.[11 30 36-57] There is considerable heterogeneity in the methods used to assess changes to the liver parenchyma. Earlier studies used CT scans and liver biopsies[37-40] and later studies used post-operative histology, reported using a variety of scoring systems.[11 30 36 41-57] Radiation exposure resulting from CT scans and the invasive nature of liver biopsies mean that neither technique is suited to providing repeated measurements, and post-operative specimens provide only a one-off assessment of the liver parenchyma. None of these assessment tools are therefore ideally suited to measuring change over time.

The link between CALIs and post-operative morbidity and mortality remains unclear with reports to date being mixed.[30 36 41-44 46-52 54-58] Some studies such as the one by Karoui et al[46] have demonstrated that CALIs increased the risk of post-operative liver failure (11% v 0%), with others such as Vauthey et al[47] demonstrating increased post-operative mortality (increased 90-day mortality in patients with steatohepatitis 14.7% v 1.6%, $P=0.001$; steatohepatitis associated with pre-operative irinotecan 20.2% v 4.4%, $P<0.001$).

Post-operative morbidity and mortality are often related to inadequate function of the remnant liver, resulting in post-operative liver failure - an important cause of mortality after partial liver resection.[14 59 60] A meta-analysis estimated the overall incidence of liver failure after hepatectomy to lie between 0.7% and 9.1%,[61] with most recent studies reporting liver

For peer review only - http://bmjopen.bmj.com/site/about/guidelines.xhtml

failure rates within this range.[62 57] The improved detection of CALIs, assessment of their impact upon post-operative outcomes and pre-operative assessment of the FLR are thus important clinical issues. Several tests and derived indices are in use, including conventional blood parameters of liver function (liver enzymes, albumin, bilirubin, INR), Child-Pugh Classification, scintigraphy, galactose elimination test and indocyanine green (ICG) clearance rate. These assessments are suboptimal as they comprise indirect measurements of liver function.

Advances in chemotherapy and hepatic surgery have expanded the pool of candidates for potentially curative hepatic resection for CLM,[7] many of whom will undergo pre-operative chemotherapy. Recognition of CALIs emphasises the need to fully assess each patient and utilise individualised planning, giving attention to the planned extent of resection, the choice of chemotherapy regimen and the potential consequences upon the hepatic parenchyma. Future challenges include the refinement of liver function assessment and the establishment of better methods for evaluation and diagnosis of post-chemotherapy liver injury.[7]

This study aims to address these challenges by utilising the following two novel techniques for assessing liver function and liver fat, measuring these repeatedly before after chemotherapy, and correlating these changes to post-operative outcomes. Performing repeated measurements over time of both liver fat deposition and liver function during and after the cessation of chemotherapy will permit better interpretation of causality and potential reversibility. This will address the mechanistic question shown in **Figure 1**: is chemotherapy-induced hepatic fat accumulation driven through liver injury as shown in Hypothesis A, or unrelated as in Hypothesis B?

For peer review only - http://bmjopen.bmj.com/site/about/guidelines.xhtml

Figure 1 (See separate image file)

Understanding these relationships and making use of the observation that pre-operative chemotherapy is a model of an extreme accelerated phenotype of liver fat deposition may also help to address some of the underlying mechanistic questions in obesity-related cancers.

The LiMAx test

The Liver Maximum capacity (LiMAx) test was developed as a novel clinical evaluation of liver function, aimed at overcoming the difficulties in accurately assessing pre-operative liver function prior to hepatic resection.[63] It is based on the metabolism of a ^{13}C-labelled substrate by the hepatocyte-specific enzyme system P450 CYP1A2, the activity of which is not influenced by drugs or genetic variations,[64] is distributed ubiquitously throughout the liver,[65] and shows a clear discrimination between normal and abnormal liver function independent of cholestasis.[66] The intravenous administration of ^{13}C-methacetin results in a significant alteration of the normal expired breath $^{13}CO_2$:$^{12}CO_2$ ratio[67] detected by breath analysis performed by an infrared spectroscopy-based FLIP device.

The LiMAx test was initially used in pre- and post-operative hepatectomy assessment, where resection of a specific percentage of functional liver volume was found to lead to an equivalent reduction in LiMAx value.[68] The LiMAx test was found to represent an accurate surrogate of liver function capacity,[68] to be an accurate predictor of post-operative liver failure and mortality,[68 63 69 70] and to be unaffected by age and gender.[71]

The LiMAx test has been evaluated as a tool with which to diagnose CALIs. LiMAx was found to be superior to other methods of assessing liver function both in detecting CALIs and their resolution, when compared to standard biochemical liver parameters (INR, bilirubin,

For peer review only - http://bmjopen.bmj.com/site/about/guidelines.xhtml

aspartate transaminase) and ICG-clearance rates.[72 73] However, it has never been used repeatedly in the setting of CLM or correlated with post-operative outcomes.

Advanced MR imaging

Traditional assessment methods failed to provide an ideal measurement tool for liver fat content (hepatic fat fraction – HFF). Ultrasonography scan (USS) and computed tomography (CT) have the ability to demonstrate fatty infiltration, however, they are unable to quantify HFF.[74-77] USS is preferred for qualitative assessment because CT sensitivity is time-dependent and protocol specific[78] and is not suitable for use in longitudinal monitoring due to its reliance on ionising radiation.[79] On the other hand, USS is low-cost, safe, and readily accessible.[79] However, USS has limited repeatability and reproducibility, and the outputs have only modest correlation with biopsy results.[78 80] Liver biopsy provides a quantitative method for the assessment of fatty liver disease, however, this is an invasive procedure hampered by sampling errors as fatty infiltrations can be unevenly distributed throughout the liver,[81-83] significant inter-observer variability[84] and potential complications such as bleeding and mortality.[85]

Magnetic resonance (MR) imaging using standard pulse sequences is insensitive to diffuse fatty infiltrations.[86-88] Techniques have been developed with the aim of further exploiting the differences in resonance frequencies between water and fat proton signals in order to assess HFF with increased accuracy, including magnetic resonance spectroscopy (MRS)[89] and the modified Dixon technique (mDixon).[90]

MRS utilises a much larger sample of the liver as compared to a typical biopsy sample, minimising the chance of sampling error, and is sensitive enough to detect small amounts of fat that may be histologically undetectable.[91] It is considered to be the optimal non-invasive method for the accurate assessment of liver fat,[92] being the technique shown to produce the most reliable and reproducible measurements.[79 81 91 93-97 98] MRS has been validated as an

For peer review only - http://bmjopen.bmj.com/site/about/guidelines.xhtml

accurate technique against the histological evaluation of fatty liver changes in multiple studies.[81 93-96 99 100] However, MRS has not achieved widespread clinical use despite the undisputed strengths of this technique. This may be due to poor availability of expertise and equipment outside the research setting.[90]

Techniques of quantitative magnetic resonance imaging have recently been introduced for the acquisition of proton density fat fraction maps covering large portions of the liver. These modified Dixon techniques (mDixon) show promising results in assessing HFF in comparisons with histological scoring systems[101-103] and correlate well with MRS.[90]

The Manchester Obesity and Cancer Research Group run a long-term research programme developing non-invasive imaging techniques to quantify liver fat. We have previously validated the use of MRS and the mDixon method against digital histological determination of fatty infiltration in post-surgical specimens, quantifying and characterising fat deposition in patients with CLM undergoing resection.[104 105] These previous studies were cross-sectional whereas the current study will evaluate changes over time in order to better assess their relationship to chemotherapy and their potential reversibility.

The proposed MR imaging protocols in the current study will be as follows:

MRS

Single volume 1H spectra are acquired using Stimulated Echo Acquisition Mode (STEAM) spectroscopy (TR = 2000 ms, TE = 10 ms, FA = 90°. 16 dynamic scans are acquired without averaging for a 15 mm3 voxel of interest (VOI) with a 32 s duration. This is repeated three times with VOIs positioned to avoid CLM and partial volume averaging with large blood vessels and bile ducts.

mDixon

For peer review only - http://bmjopen.bmj.com/site/about/guidelines.xhtml

A six-echo 3D gradient echo sequence is used, with first TE at 0.92 ms and a delta of 0.7 ms (TR = 5.3 ms, FA = 5°). Full liver coverage is acquired with 67 axial slices and 3 mm2 in-plane and 6 mm through plane resolution, interpolated to 2 mm2 in-plane and 3 mm through plane. Parallel acquisition used with a SENSE factor of 1.5 in the AP plane. The full volume is acquired in a single breath hold for a duration of 12 seconds. Quantitative fat fraction maps are calculated using a dedicated scanner software package. A representative fat fraction for each subject is calculated using the average from three liver-tissue regions of interest positioned on a central slice, using the same criteria as for the spectroscopy.

AIMS

To prospectively assess the changes in liver function and fat resulting from the administration of pre-operative chemotherapy prior to hepatic resection for colorectal liver metastases (Change in Liver Function and Fat – the CLiFF study). These changes will be assessed repeatedly using the novel techniques of the LiMAx test and advanced MR imaging as demonstrated in **Figure 2**, then correlated with standard assessments and post-operative outcomes. To our knowledge, there is no prospective evaluation of this type to-date.

Figure 2 – Study Participant Timeline (See separate image file)

Primary outcome measures

1. Change in liver function measured by the LiMAx test performed using the FLIP device, comparing baseline measurements with measurements during and after chemotherapy.
2. Change in liver fat as a percentage (Hepatic Fat Fraction – HFF) measured by advanced Magnetic Resonance imaging (utilising both Magnetic Resonance Spectroscopy and the modified Dixon method), comparing baseline measurements with measurements during and after chemotherapy.

Secondary outcome measures

For peer review only - http://bmjopen.bmj.com/site/about/guidelines.xhtml

1) Changes in routinely collected liver enzymes by blood testing
2) Digital histological quantification of intrahepatic fat
3) Correlations with BMI and image-determined anthropometric measurements e.g. subcutaneous adipose tissue (SAT), visceral adipose tissue volumes (VAT) and muscle mass
4) Grade of steatosis scores (NAS-CRN) from routine histology reporting
5) Post-operative outcomes, including peri-operative mortality and morbidity (Clavien-Dindo classification, Comprehensive Complication Index, International Study Group for Liver Surgery complications).
6) Change in health-related quality of life by measurement of EQ-5D score

METHODS

Study design

This is a non-randomised prospective observational study of up to 35 patients with CLM.

Study setting

The study is sponsored by the University of Manchester and will be performed at The Christie NHS Foundation Trust and the University of Manchester Wolfson Molecular Imaging Centre. Participant identification will take place at the joint Christie-Manchester Foundation Trust hepatopancreatobiliary (HPB) multidisciplinary team (MDT) meeting. Team members will screen all patients for whom an MDT decision is made that a course of pre-operative chemotherapy is indicated prior to potentially curative hepatic resection of CLM.

Institutions

From October 6, 2014, a single Improving Outcomes Guidance-compliant HPB Service has been located at the Manchester Royal Infirmary site at Manchester University NHS Foundation Trust. It hosts the regional specialist HPB MDT meeting. All patients selected to

For peer review only - http://bmjopen.bmj.com/site/about/guidelines.xhtml

undergo neoadjuvant chemotherapy prior to hepatic resection from this MDT will receive this treatment via the oncology service delivered from the Christie Hospital.

The Wolfson Molecular Imaging Centre is part of the University of Manchester. This centre provides state-of-the art imaging facilities and radiological expertise in the advanced imaging techniques of MRS and mDixon. The Philips 1.5T scanner at WMIC was upgraded to provide mDixon in addition to the established MRS.

Investigators

The CLiFF study is led by a dedicated clinical research fellow, as part of a University-registered thesis. The supervisory team provide the relevant expertise in surgical cancer research, hepatobiliary surgery, medical oncology and imaging science, with additional research and statistical support available as required.

Inclusion criteria

Patients with liver metastases of colorectal origin, undergoing pre-operative standard chemotherapy, with the following criteria:

1. Histologically verified adenocarcinoma of the colon or rectum with radiological evidence of potentially resectable liver metastases;
2. No evidence of unresectable non-hepatic metastatic disease;
3. Adequate haematological and hepatic function defined by: Hb $\geq$ 10g/dl, WBC $\geq$ 3.0 x 10^9/L, ANC $\geq$ 1.5 x 10^9/L, platelet count $\geq$ 100,000/ mm^3, total bilirubin < 30 μmol/L, serum AST, ALT and alkaline phosphatase $\leq$ 5 x upper limit of normal;
4. Written informed consent and able to attend long-term follow-up.

Exclusion criteria

For peer review only - http://bmjopen.bmj.com/site/about/guidelines.xhtml

1. Presence of a medical or psychiatric condition that impairs their ability to give informed consent
2. Presence of any other serious uncontrolled medical conditions
3. Evidence of unresectable non-hepatic metastatic disease

Patient identification

Patients will be screened for inclusion initially via discussion at the specialist HPB MDT meeting and by referral to the Medical Oncology service. Subsequent to an MDT decision that pre-operative chemotherapy is indicated prior to potentially curative resection of colorectal liver metastases, the CLiFF study team will screen for the remaining inclusion criteria. Potential participants will be invited to join the study via the provision of verbal and written information regarding the CLiFF study. Confirmation of the wish to participate will subsequently be confirmed and informed consent taken prior to beginning chemotherapy.

Data collection

Participants will undergo assessment of liver function by LiMAx testing and HFF by advanced MR imaging at the times outlined in the study schedule shown in Appendix 1 and 2. All clinicians involved in the medical and surgical treatment of the study participants will be blinded to the results of the LiMAx testing and MR imaging. The study MR images will not be reported by a clinical radiologist. All other aspects of treatment will remain unchanged and proceed as per standard care.

Data collection will include LiMAx tests and MR results, in addition to the results from all other assessments outlined in Appendix 2. The LiMAx tests and MR scans will be performed directly by the clinical research fellow. The remaining information will be taken from the medical notes, comprising the results from routine oncology outpatient assessments, the post-operative histology report and the post-operative outcomes following hepatic resection.

For peer review only - http://bmjopen.bmj.com/site/about/guidelines.xhtml

Informed consent will be taken for the research team to access this information from the medical records.

Data management

The NHS code of confidentiality will be observed, with only the clinical care team and team directly involved in the research having access to any identifiable data. All data will be pseudonymised with a unique identifier and stored in a secure encrypted database. A key to patient identifiers will be stored in a separate encrypted document. Data stored by the research team will therefore not contain patient identifiers. Data will be collected and stored in accordance with the General Data Protection Regulation (GDPR) and Data Protection Act 2018. The University of Manchester, as Data Controller for this study, takes responsibility for the protection of any personal information collected by this study. All researchers will be appropriately trained in data protection.

Statistical analysis & power calculation

Our previous work reported a mean (±sd) Hepatic Fat Fraction of 4 (±2) %.[105] Following chemotherapy, we estimate at least a 50% increase in HFF i.e. increase to mean (sd) = 6 ± 2.5%. We have planned a recruitment of at least 35 patients. As attrition (for multiple reasons) is common in this type of study, of the 35 patients, we conservatively estimate that 25 patients will have complete data from at least two MR evaluations. The resultant power is 98% (alpha: 0.05; within-person correlation: 0.7).

For the LiMAx testing, which is more frequent in the study schedule, we estimate at least 32 patients (10% attrition rate) will have complete data from two LiMAx tests. Based on the Lock et al [2017] study in patients undergoing pre-resection chemotherapy[73] [mean (±sd): 340 ± 95 vs. 391 ± 82 µg/kg/h], the resultant power is 84% (alpha: 0.05; within-person correlation: 0.7).

For peer review only - http://bmjopen.bmj.com/site/about/guidelines.xhtml

Statistical support will be provided where required by the University of Manchester Cancer Data Science Team. The main analysis will focus on intra-patient change in both liver function (measured by the LiMAx test) and hepatic fat fraction (measured by advanced MR imaging) which occurs subsequent to chemotherapy, and their recovery prior to surgery. These changes will be related to subsequent clinical course, CT volumetry and histopathological analysis of the resected liver. We will explore the use of ANOVA methods and mixed-effects methods to account for within-person correlations. For each time point, standard approaches to categorical (chi-squared test) and continuous (Mann-Whitney U test) variables will be used. Correlation matrices will be constructed to assess relationships between LiMAx and HFF with other continuous variables using Spearman's Rank Correlation Coefficient.

To account for multiple testing, a p-value of <0.01 will be considered to indicate statistical significance. Predictors of HFF at each time point will be explored using multivariable linear regression models. To reduce the anticipated right skewness of the distributions, logarithmic transformation will be explored.

Because of the large number of variables and the anticipated high levels of correlations, we will use factor-cluster methods. There will be at least five clusters: patient-related factors; routine blood measures; CT-derived anthropometrics; LiMAx readouts; and MR readouts. Separate models will be developed for each cluster and significant (p <0.05) variables selected for the final model.

Anticipated recruitment

At the specialist HPB MDT, there are approximately 120 resections per year performed for CLM. Approximately half these patients will receive pre-operative chemotherapy. Of these 60 patients, 40 will have their chemotherapy delivered at the oncology centre and will be potentially eligible for recruitment. Over the past 4 years, the numbers of liver resections

For peer review only - http://bmjopen.bmj.com/site/about/guidelines.xhtml

performed for CLM has consistently increased year on year. We therefore anticipate that the recruitment target of 35 patients to be recruited and followed up within two years is achievable.

Quality assurance

The quality of this study has been assessed by the following means:

- Departmental review within the University of Manchester
- Peer review by professionals with relevant expertise (Clinical trialists, statisticians, surgeons, oncologists and imaging scientists)
- Review by the Research Practice Governance Team at the University of Manchester (Sponsor Institution) and the Research & Development team at the Christie NHS Foundation Trust (host)
- Peer review arranged by the North West Innovation Service as part of a successful competitive funding application
- Review by the Royal College of Surgeons research department as part of a successful competitive application for a research fellowship
- Independent peer review as part of the registration process for ClinicalTrials.gov, a publicly accessible database of worldwide research studies, maintained by the National Library of Medicine at the National Institutes for Health (US) (NCT03562234)

For peer review only

Study Timetable

The anticipated study timetable is outlined in Table 1.

Table 1: Study Timetable

Time Period	Date
Study Set-Up and Approvals	01/04/2018 - 01/09/2018
Participant Recruitment	01/10/2018 - 01/10/2019
Data collection	01/10/2019- 01/03/2020

For peer review only - http://bmjopen.bmj.com/site/about/guidelines.xhtml

Data Analysis and Write-Up	01/03/2020 – 31/05/2020

ETHICS AND DISSEMINATION

Ethical approval

Ethical approval for this study was granted by the National Health Service North West Research Ethics Committee following the meeting held on 27th July 2018 (REC Reference 18/NW/0531).

Registration

This study was registered, reviewed and approved by the Health Research Authority (HRA). It has been registered with the sponsor institution (University of Manchester) and approved by their Research Practice Governance Team. The host institution (Christie NHS Foundation Trust) Research & Development team have issued approval of their capability and capacity to host this study. The study was registered online at www.clinicaltrials.gov, registration number NCT03562234.

Patient and Public Involvement

The Public Programmes Team at the Manchester University NHS Foundation Trust hosted a discussion group for the CLiFF Study with six members of the NIHR Manchester Biomedical Research Centre funded Cancer Research Advisory Panel on 24/05/2018. The six attending members were all patients/carers with personal experience of CLM treatment. This event was attended by the Principle Investigator to actively seek and hear the views of the patient and public representatives. The group unanimously found that this area of research was important, found no feasibility or acceptability issues with the study and thought it likely that they would take part if asked. The following modifications were made in response to the session:

1) **Amendments to Participant Information Sheet:**

For peer review only - http://bmjopen.bmj.com/site/about/guidelines.xhtml

All group suggestions were incorporated, including the requested addition of a clearly highlighted opening statement explaining that treatment would not be modified as the result of participating in this study.

2) **Training to use long-term intravenous access devices:**

Patients who had previously undergone pre-operative chemotherapy for CLM expressed frustration that whilst many of them had long-term intravenous access devices for the duration of their treatment, inadequate training to access these devices resulted in additional exposure to needles. They requested that where possible, these long-term devices be used for the intravenous injection component of the LiMAx test. In response to this request, the Principal Investigator underwent additional training to ensure that long-term intravenous access devices could be utilised for the LiMAx tests performed in this study.

One patient member of the Cancer Research Advisory Panel contributed substantially and subsequently became a co-author of this protocol paper (CB).

Dissemination

This study will be submitted for presentation at national or international surgical conferences. Manuscript(s) will be prepared following close of the study.

Figure Legends

Figure 1: Direct Acyclic Graph

Figure 2: Study Participant Timeline

Authorship contributions

For peer review only - http://bmjopen.bmj.com/site/about/guidelines.xhtml

For peer review only

The study concept was conceived by JWV, DOR and AGR. The present protocol was written by DOR, AGR and KLP. All authors contributed to the revision and preparation of the manuscript.

This research has received funding from the following sources:

Humedics GMBH; National Institute for Health Research Manchester Biomedical Research Centre; North West NHS Innovation service; The Christie NHS Foundation Trust charitable funds; The Royal College of Surgeons of England Fellowship scheme

Conflicts of interest: AGR reports personal fees from Merck Serona, personal fees from Janssen-Cilag, grants and personal fees from Sanofi Pasteur MSD, outside the submitted work. The remaining authors declare no conflicts.

For peer review only

For peer review only - http://bmjopen.bmj.com/site/about/guidelines.xhtml

References

1. CRUK. Cancer Research UK - Bowel Cancer Statistics 2018 [Available from: http://www.cancerresearchuk.org/health-professional/cancer-statistics/statistics-by-cancer-type/bowel-cancer accessed 2/04/2018.
2. Fiorentini G, Sarti D, Aliberti C, et al. Multidisciplinary approach of colorectal cancer liver metastases. *World J Clin Oncol* 2017;8(3):190-202. doi: 10.5306/wjco.v8.i3.190 [published Online First: 2017/06/24]
3. Manfredi S, Lepage C, Hatem C, et al. Epidemiology and management of liver metastases from colorectal cancer. *Ann Surg* 2006;244(2):254-9. doi: 10.1097/01.sla.0000217629.94941.cf [published Online First: 2006/07/22]
4. Nordlinger B, Van Cutsem E, Gruenberger T, et al. Combination of surgery and chemotherapy and the role of targeted agents in the treatment of patients with colorectal liver metastases: recommendations from an expert panel. *Ann Oncol* 2009;20(6):985-92. doi: 10.1093/annonc/mdn735 [published Online First: 2009/01/21]
5. Adam R, De Gramont A, Figueras J, et al. The oncosurgery approach to managing liver metastases from colorectal cancer: a multidisciplinary international consensus. *Oncologist* 2012;17(10):1225-39. doi: 10.1634/theoncologist.2012-0121 [published Online First: 2012/09/11]
6. Renehan AG, Egger M, Saunders MP, et al. Impact on survival of intensive follow up after curative resection for colorectal cancer: systematic review and meta-analysis of randomised trials. *BMJ* 2002;324(7341):813. [published Online First: 2002/04/06]
7. Zorzi D, Laurent A, Pawlik TM, et al. Chemotherapy-associated hepatotoxicity and surgery for colorectal liver metastases. *Br J Surg* 2007;94(3):274-86. doi: 10.1002/bjs.5719 [published Online First: 2007/02/23]
8. Schroeder RA, Marroquin CE, Bute BP, et al. Predictive indices of morbidity and mortality after liver resection. *Ann Surg* 2006;243(3):373-9. doi: 10.1097/01.sla.0000201483.95911.08 [published Online First: 2006/02/24]
9. Dinant S, de Graaf W, Verwer BJ, et al. Risk assessment of posthepatectomy liver failure using hepatobiliary scintigraphy and CT volumetry. *J Nucl Med* 2007;48(5):685-92. doi: 10.2967/jnumed.106.038430 [published Online First: 2007/05/04]
10. Erdogan D, Heijnen BH, Bennink RJ, et al. Preoperative assessment of liver function: a comparison of 99mTc-Mebrofenin scintigraphy with indocyanine green clearance test. *Liver Int* 2004;24(2):117-23. doi: 10.1111/j.1478-3231.2004.0901.x [published Online First: 2004/04/14]
11. Fernandez FG, Ritter J, Goodwin JW, et al. Effect of steatohepatitis associated with irinotecan or oxaliplatin pretreatment on resectability of hepatic colorectal metastases. *J Am Coll Surg* 2005;200(6):845-53. doi: 10.1016/j.jamcollsurg.2005.01.024 [published Online First: 2005/06/01]
12. Schindl MJ, Redhead DN, Fearon KC, et al. The value of residual liver volume as a predictor of hepatic dysfunction and infection after major liver resection. *Gut* 2005;54(2):289-96. doi: 10.1136/gut.2004.046524 [published Online First: 2005/01/14]
13. Yigitler C, Farges O, Kianmanesh R, et al. The small remnant liver after major liver resection: how common and how relevant? *Liver Transpl* 2003;9(9):S18-25. doi: 10.1053/jlts.2003.50194 [published Online First: 2003/08/28]
14. Farges O, Belghiti J, Kianmanesh R, et al. Portal vein embolization before right hepatectomy: prospective clinical trial. *Ann Surg* 2003;237(2):208-17. doi: 10.1097/01.SLA.0000048447.16651.7B [published Online First: 2003/02/01]
15. Wakabayashi H, Ishimura K, Okano K, et al. Application of preoperative portal vein embolization before major hepatic resection in patients with normal or abnormal liver parenchyma. *Surgery* 2002;131(1):26-33. [published Online First: 2002/01/29]

For peer review only - http://bmjopen.bmj.com/site/about/guidelines.xhtml

16. Folprecht G, Gruenberger T, Bechstein WO, et al. Tumour response and secondary resectability of colorectal liver metastases following neoadjuvant chemotherapy with cetuximab: the CELIM randomised phase 2 trial. *Lancet Oncol* 2010;11(1):38-47. doi: 10.1016/S1470-2045(09)70330-4 [published Online First: 2009/11/28]
17. Fausto N, Campbell JS, Riehle KJ. Liver regeneration. *Hepatology* 2006;43(2 Suppl 1):S45-53. doi: 10.1002/hep.20969 [published Online First: 2006/02/01]
18. Ikeda Y, Matsumata T, Takenaka K, et al. Preliminary report of tumor metastasis during liver regeneration after hepatic resection in rats. *Eur J Surg Oncol* 1995;21(2):188-90. [published Online First: 1995/04/01]
19. Nagashima I, Takada T, Adachi M, et al. Proposal of criteria to select candidates with colorectal liver metastases for hepatic resection: comparison of our scoring system to the positive number of risk factors. *World J Gastroenterol* 2006;12(39):6305-9. [published Online First: 2006/10/31]
20. Schneider PD. Preoperative assessment of liver function. *Surg Clin North Am* 2004;84(2):355-73. doi: 10.1016/S0039-6109(03)00224-X [published Online First: 2004/04/06]
21. Khan AZ, Toogood GJ. The case for adjuvant chemotherapy. Against. *Ann R Coll Surg Engl* 2008;90(6):454-5. [published Online First: 2008/09/09]
22. Morris EJ, Forman D, Thomas JD, et al. Surgical management and outcomes of colorectal cancer liver metastases. *Br J Surg* 2010;97(7):1110-8. doi: 10.1002/bjs.7032 [published Online First: 2010/07/16]
23. Mattar RE, Al-Alem F, Simoneau E, et al. Preoperative selection of patients with colorectal cancer liver metastasis for hepatic resection. *World J Gastroenterol* 2016;22(2):567-81. doi: 10.3748/wjg.v22.i2.567 [published Online First: 2016/01/27]
24. Abdalla EK, Vauthey JN, Ellis LM, et al. Recurrence and outcomes following hepatic resection, radiofrequency ablation, and combined resection/ablation for colorectal liver metastases. *Ann Surg* 2004;239(6):818-25; discussion 25-7. [published Online First: 2004/05/29]
25. Choti MA, Sitzmann JV, Tiburi MF, et al. Trends in long-term survival following liver resection for hepatic colorectal metastases. *Ann Surg* 2002;235(6):759-66. [published Online First: 2002/05/30]
26. Fernandez FG, Drebin JA, Linehan DC, et al. Five-year survival after resection of hepatic metastases from colorectal cancer in patients screened by positron emission tomography with F-18 fluorodeoxyglucose (FDG-PET). *Ann Surg* 2004;240(3):438-47; discussion 47-50. [published Online First: 2004/08/21]
27. Pawlik TM, Scoggins CR, Zorzi D, et al. Effect of surgical margin status on survival and site of recurrence after hepatic resection for colorectal metastases. *Ann Surg* 2005;241(5):715-22, discussion 22-4. [published Online First: 2005/04/26]
28. Simmonds PC, Primrose JN, Colquitt JL, et al. Surgical resection of hepatic metastases from colorectal cancer: a systematic review of published studies. *Br J Cancer* 2006;94(7):982-99. doi: 10.1038/sj.bjc.6603033 [published Online First: 2006/03/16]
29. Nordlinger B, Van Cutsem E, Rougier P, et al. Does chemotherapy prior to liver resection increase the potential for cure in patients with metastatic colorectal cancer? A report from the European Colorectal Metastases Treatment Group. *Eur J Cancer* 2007;43(14):2037-45. doi: 10.1016/j.ejca.2007.07.017 [published Online First: 2007/09/04]
30. Adam R, Delvart V, Pascal G, et al. Rescue surgery for unresectable colorectal liver metastases downstaged by chemotherapy: a model to predict long-term survival. *Ann Surg* 2004;240(4):644-57; discussion 57-8. [published Online First: 2004/09/24]
31. Adam R, Pascal G, Castaing D, et al. Tumor progression while on chemotherapy: a contraindication to liver resection for multiple colorectal metastases? *Ann Surg* 2004;240(6):1052-61; discussion 61-4. [published Online First: 2004/12/01]
32. Abdalla EK, Barnett CC, Doherty D, et al. Extended hepatectomy in patients with hepatobiliary malignancies with and without preoperative portal vein embolization. *Arch Surg* 2002;137(6):675-80; discussion 80-1. [published Online First: 2002/06/07]

For peer review only - http://bmjopen.bmj.com/site/about/guidelines.xhtml

33. Jaeck D, Oussoultzoglou E, Rosso E, et al. A two-stage hepatectomy procedure combined with portal vein embolization to achieve curative resection for initially unresectable multiple and bilobar colorectal liver metastases. *Ann Surg* 2004;240(6):1037-49; discussion 49-51. [published Online First: 2004/12/01]
34. Adam R, Avisar E, Ariche A, et al. Five-year survival following hepatic resection after neoadjuvant therapy for nonresectable colorectal. *Ann Surg Oncol* 2001;8(4):347-53. [published Online First: 2001/05/16]
35. Masi G, Cupini S, Marcucci L, et al. Treatment with 5-fluorouracil/folinic acid, oxaliplatin, and irinotecan enables surgical resection of metastases in patients with initially unresectable metastatic colorectal cancer. *Ann Surg Oncol* 2006;13(1):58-65. doi: 10.1245/ASO.2006.03.094 [published Online First: 2005/12/24]
36. Nordlinger B, Sorbye H, Glimelius B, et al. Perioperative chemotherapy with FOLFOX4 and surgery versus surgery alone for resectable liver metastases from colorectal cancer (EORTC Intergroup trial 40983): a randomised controlled trial. *Lancet* 2008;371(9617):1007-16. doi: 10.1016/S0140-6736(08)60455-9 [published Online First: 2008/03/25]
37. Zeiss J, Merrick HW, Savolaine ER, et al. Fatty liver change as a result of hepatic artery infusion chemotherapy. *Am J Clin Oncol* 1990;13(2):156-60. [published Online First: 1990/04/01]
38. Moertel CG, Fleming TR, Macdonald JS, et al. Hepatic toxicity associated with fluorouracil plus levamisole adjuvant therapy. *J Clin Oncol* 1993;11(12):2386-90. doi: 10.1200/JCO.1993.11.12.2386 [published Online First: 1993/12/01]
39. Sorensen P, Edal AL, Madsen EL, et al. Reversible hepatic steatosis in patients treated with interferon alfa-2a and 5-fluorouracil. *Cancer* 1995;75(10):2592-6. [published Online First: 1995/05/15]
40. Peppercorn PD, Reznek RH, Wilson P, et al. Demonstration of hepatic steatosis by computerized tomography in patients receiving 5-fluorouracil-based therapy for advanced colorectal cancer. *Br J Cancer* 1998;77(11):2008-11. [published Online First: 1998/07/17]
41. Behrns KE, Tsiotos GG, DeSouza NF, et al. Hepatic steatosis as a potential risk factor for major hepatic resection. *J Gastrointest Surg* 1998;2(3):292-8. [published Online First: 1998/12/08]
42. Belghiti J, Hiramatsu K, Benoist S, et al. Seven hundred forty-seven hepatectomies in the 1990s: an update to evaluate the actual risk of liver resection. *J Am Coll Surg* 2000;191(1):38-46. [published Online First: 2000/07/18]
43. Parikh AA, Gentner B, Wu TT, et al. Perioperative complications in patients undergoing major liver resection with or without neoadjuvant chemotherapy. *J Gastrointest Surg* 2003;7(8):1082-8. [published Online First: 2003/12/17]
44. Kooby DA, Fong Y, Suriawinata A, et al. Impact of steatosis on perioperative outcome following hepatic resection. *J Gastrointest Surg* 2003;7(8):1034-44. [published Online First: 2003/12/17]
45. Rubbia-Brandt L, Audard V, Sartoretti P, et al. Severe hepatic sinusoidal obstruction associated with oxaliplatin-based chemotherapy in patients with metastatic colorectal cancer. *Ann Oncol* 2004;15(3):460-6. [published Online First: 2004/03/05]
46. Karoui M, Penna C, Amin-Hashem M, et al. Influence of preoperative chemotherapy on the risk of major hepatectomy for colorectal liver metastases. *Ann Surg* 2006;243(1):1-7. [published Online First: 2005/12/24]
47. Vauthey JN, Pawlik TM, Ribero D, et al. Chemotherapy regimen predicts steatohepatitis and an increase in 90-day mortality after surgery for hepatic colorectal metastases. *J Clin Oncol* 2006;24(13):2065-72. doi: 10.1200/jco.2005.05.3074 [published Online First: 2006/05/02]
48. Pawlik TM, Olino K, Gleisner AL, et al. Preoperative chemotherapy for colorectal liver metastases: impact on hepatic histology and postoperative outcome. *J Gastrointest Surg* 2007;11(7):860-8. doi: 10.1007/s11605-007-0149-4 [published Online First: 2007/05/12]

For peer review only - http://bmjopen.bmj.com/site/about/guidelines.xhtml

49. Hewes JC, Dighe S, Morris RW, et al. Preoperative chemotherapy and the outcome of liver resection for colorectal metastases. *World J Surg* 2007;31(2):353-64; discussion 65-6. doi: 10.1007/s00268-006-0103-8 [published Online First: 2007/01/16]
50. Nakano H, Oussoultzoglou E, Rosso E, et al. Sinusoidal injury increases morbidity after major hepatectomy in patients with colorectal liver metastases receiving preoperative chemotherapy. *Ann Surg* 2008;247(1):118-24. doi: 10.1097/SLA.0b013e31815774de [published Online First: 2007/12/25]
51. Hewes J. Re: Preoperative chemotherapy and the outcome of liver resection for colorectal metastases. *World J Surg* 2007;31(9):1902; author reply 03. doi: 10.1007/s00268-007-9065-8 [published Online First: 2007/05/10]
52. Soubrane O, Brouquet A, Zalinski S, et al. Predicting high grade lesions of sinusoidal obstruction syndrome related to oxaliplatin-based chemotherapy for colorectal liver metastases: correlation with post-hepatectomy outcome. *Ann Surg* 2010;251(3):454-60. doi: 10.1097/SLA.0b013e3181c79403 [published Online First: 2010/02/18]
53. Tamandl D, Klinger M, Eipeldauer S, et al. Sinusoidal obstruction syndrome impairs long-term outcome of colorectal liver metastases treated with resection after neoadjuvant chemotherapy. *Ann Surg Oncol* 2011;18(2):421-30. doi: 10.1245/s10434-010-1317-4 [published Online First: 2010/09/17]
54. Wolf PS, Park JO, Bao F, et al. Preoperative chemotherapy and the risk of hepatotoxicity and morbidity after liver resection for metastatic colorectal cancer: a single institution experience. *J Am Coll Surg* 2013;216(1):41-9. doi: 10.1016/j.jamcollsurg.2012.08.030 [published Online First: 2012/10/09]
55. Parkin E, O'Reilly DA, Adam R, et al. Equivalent survival in patients with and without steatosis undergoing resection for colorectal liver metastases following pre-operative chemotherapy. *Eur J Surg Oncol* 2014 doi: 10.1016/j.ejso.2014.07.040 [published Online First: 2014/09/06]
56. Vigano L, Capussotti L, De Rosa G, et al. Liver resection for colorectal metastases after chemotherapy: impact of chemotherapy-related liver injuries, pathological tumor response, and micrometastases on long-term survival. *Ann Surg* 2013;258(5):731-40; discussion 41-2. doi: 10.1097/SLA.0b013e3182a6183e [published Online First: 2013/09/21]
57. Vigano L, De Rosa G, Toso C, et al. Reversibility of chemotherapy-related liver injury. *J Hepatol* 2017;67(1):84-91. doi: 10.1016/j.jhep.2017.02.031 [published Online First: 2017/03/13]
58. Welsh FK, Tilney HS, Tekkis PP, et al. Safe liver resection following chemotherapy for colorectal metastases is a matter of timing. *Br J Cancer* 2007;96(7):1037-42. doi: 10.1038/sj.bjc.6603670 [published Online First: 2007/03/14]
59. Poon RT, Fan ST, Lo CM, et al. Extended hepatic resection for hepatocellular carcinoma in patients with cirrhosis: is it justified? *Ann Surg* 2002;236(5):602-11. doi: 10.1097/01.SLA.0000033038.38956.5E [published Online First: 2002/11/01]
60. Farges O, Malassagne B, Flejou JF, et al. Risk of major liver resection in patients with underlying chronic liver disease: a reappraisal. *Ann Surg* 1999;229(2):210-5. [published Online First: 1999/02/19]
61. van den Broek MA, Olde Damink SW, Dejong CH, et al. Liver failure after partial hepatic resection: definition, pathophysiology, risk factors and treatment. *Liver Int* 2008;28(6):767-80. doi: 10.1111/j.1478-3231.2008.01777.x [published Online First: 2008/07/24]
62. Ryan P, Nanji S, Pollett A, et al. Chemotherapy-induced liver injury in metastatic colorectal cancer: semiquantitative histologic analysis of 334 resected liver specimens shows that vascular injury but not steatohepatitis is associated with preoperative chemotherapy. *Am J Surg Pathol* 2010;34(6):784-91. doi: 10.1097/PAS.0b013e3181dc242c [published Online First: 2010/04/28]
63. Stockmann M, Lock JF, Malinowski M, et al. The LiMAx test: a new liver function test for predicting postoperative outcome in liver surgery. *HPB (Oxford)* 2010;12(2):139-46. doi: 10.1111/j.1477-2574.2009.00151.x [published Online First: 2010/05/25]

For peer review only - http://bmjopen.bmj.com/site/about/guidelines.xhtml

64. Jiang Z, Dragin N, Jorge-Nebert LF, et al. Search for an association between the human CYP1A2 genotype and CYP1A2 metabolic phenotype. *Pharmacogenet Genomics* 2006;16(5):359-67. doi: 10.1097/01.fpc.0000204994.99429.46 [published Online First: 2006/04/13]
65. Palmer CN, Coates PJ, Davies SE, et al. Localization of cytochrome P-450 gene expression in normal and diseased human liver by in situ hybridization of wax-embedded archival material. *Hepatology* 1992;16(3):682-7. [published Online First: 1992/09/01]
66. George J, Murray M, Byth K, et al. Differential alterations of cytochrome P450 proteins in livers from patients with severe chronic liver disease. *Hepatology* 1995;21(1):120-8. [published Online First: 1995/01/01]
67. Schoeller DA, Schneider JF, Solomons NW, et al. Clinical diagnosis with the stable isotope 13C in CO2 breath tests: methodology and fundamental considerations. *J Lab Clin Med* 1977;90(3):412-21. [published Online First: 1977/09/01]
68. Stockmann M, Lock JF, Riecke B, et al. Prediction of postoperative outcome after hepatectomy with a new bedside test for maximal liver function capacity. *Ann Surg* 2009;250(1):119-25. doi: 10.1097/SLA.0b013e3181ad85b5 [published Online First: 2009/06/30]
69. Lock JF, Malinowski M, Seehofer D, et al. Function and volume recovery after partial hepatectomy: influence of preoperative liver function, residual liver volume, and obesity. *Langenbecks Arch Surg* 2012;397(8):1297-304. doi: 10.1007/s00423-012-0972-2 [published Online First: 2012/06/26]
70. Jara M, Reese T, Malinowski M, et al. Reductions in post-hepatectomy liver failure and related mortality after implementation of the LiMAx algorithm in preoperative work-up: a single-centre analysis of 1170 hepatectomies of one or more segments. *HPB (Oxford)* 2015;17(7):651-8. doi: 10.1111/hpb.12424 [published Online First: 2015/06/11]
71. Jara M, Bednarsch J, Valle E, et al. Reliable assessment of liver function using LiMAx. *J Surg Res* 2015;193(1):184-9. doi: 10.1016/j.jss.2014.07.041 [published Online First: 2014/08/26]
72. Jara M, Bednarsch J, Malinowski M, et al. Effects of oxaliplatin-based chemotherapy on liver function--an analysis of impact and functional recovery using the LiMAx test. *Langenbecks Arch Surg* 2016;401(1):33-41. doi: 10.1007/s00423-015-1352-5 [published Online First: 2015/10/29]
73. Lock JF, Westphal T, Rubin T, et al. LiMAx Test Improves Diagnosis of Chemotherapy-Associated Liver Injury Before Resection of Colorectal Liver Metastases. *Ann Surg Oncol* 2017;24(9):2447-55. doi: 10.1245/s10434-017-5887-2 [published Online First: 2017/05/19]
74. Foster KJ, Dewbury KC, Griffith AH, et al. The accuracy of ultrasound in the detection of fatty infiltration of the liver. *Br J Radiol* 1980;53(629):440-2. doi: 10.1259/0007-1285-53-629-440 [published Online First: 1980/05/01]
75. Bydder GM, Kreel L, Chapman RW, et al. Accuracy of computed tomography in diagnosis of fatty liver. *Br Med J* 1980;281(6247):1042. [published Online First: 1980/10/18]
76. Piekarski J, Goldberg HI, Royal SA, et al. Difference between liver and spleen CT numbers in the normal adult: its usefulness in predicting the presence of diffuse liver disease. *Radiology* 1980;137(3):727-9. doi: 10.1148/radiology.137.3.6934563 [published Online First: 1980/12/01]
77. Meek DR, Mills PR, Gray HW, et al. A comparison of computed tomography, ultrasound and scintigraphy in the diagnosis of alcoholic liver disease. *Br J Radiol* 1984;57(673):23-7. doi: 10.1259/0007-1285-57-673-23 [published Online First: 1984/01/01]
78. Graif M, Yanuka M, Baraz M, et al. Quantitative estimation of attenuation in ultrasound video images: correlation with histology in diffuse liver disease. *Invest Radiol* 2000;35(5):319-24. [published Online First: 2000/05/10]

For peer review only - http://bmjopen.bmj.com/site/about/guidelines.xhtml

79. Reeder SB, Cruite I, Hamilton G, et al. Quantitative Assessment of Liver Fat with Magnetic Resonance Imaging and Spectroscopy. *J Magn Reson Imaging* 2011;34(4):spcone. doi: 10.1002/jmri.22775 [published Online First: 2011/10/26]
80. Davies RJ, Saverymuttu SH, Fallowfield M, et al. Paradoxical lack of ultrasound attenuation with gross fatty change in the liver. *Clin Radiol* 1991;43(6):393-6. [published Online First: 1991/06/01]
81. Thomsen C, Becker U, Winkler K, et al. Quantification of liver fat using magnetic resonance spectroscopy. *Magn Reson Imaging* 1994;12(3):487-95. [published Online First: 1994/01/01]
82. Hatfield MK, Beres RA, Sane SS, et al. Percutaneous imaging-guided solid organ core needle biopsy: coaxial versus noncoaxial method. *AJR Am J Roentgenol* 2008;190(2):413-7. doi: 10.2214/AJR.07.2676 [published Online First: 2008/01/24]
83. Ratziu V, Charlotte F, Heurtier A, et al. Sampling variability of liver biopsy in nonalcoholic fatty liver disease. *Gastroenterology* 2005;128(7):1898-906. [published Online First: 2005/06/09]
84. El-Badry AM, Breitenstein S, Jochum W, et al. Assessment of hepatic steatosis by expert pathologists: the end of a gold standard. *Ann Surg* 2009;250(5):691-7. doi: 10.1097/SLA.0b013e3181bcd6dd [published Online First: 2009/10/07]
85. Bravo AA, Sheth SG, Chopra S. Liver biopsy. *N Engl J Med* 2001;344(7):495-500. doi: 10.1056/NEJM200102153440706 [published Online First: 2001/02/15]
86. Haaga JR. Magnetic resonance imaging of the liver. *Radiol Clin North Am* 1984;22(4):879-90. [published Online First: 1984/12/01]
87. Stark DD, Moss AA, Goldberg HI. Nuclear magnetic resonance of the liver, spleen, and pancreas. *Cardiovasc Intervent Radiol* 1986;8(5-6):329-41. [published Online First: 1986/01/01]
88. Kanzer GK, Weinreb JC. Magnetic resonance imaging of diseases of the liver and biliary system. *Radiol Clin North Am* 1991;29(6):1259-84. [published Online First: 1991/11/01]
89. Lee JK, Dixon WT, Ling D, et al. Fatty infiltration of the liver: demonstration by proton spectroscopic imaging. Preliminary observations. *Radiology* 1984;153(1):195-201. doi: 10.1148/radiology.153.1.6089264 [published Online First: 1984/10/01]
90. Kukuk GM, Hittatiya K, Sprinkart AM, et al. Comparison between modified Dixon MRI techniques, MR spectroscopic relaxometry, and different histologic quantification methods in the assessment of hepatic steatosis. *Eur Radiol* 2015;25(10):2869-79. doi: 10.1007/s00330-015-3703-6 [published Online First: 2015/04/24]
91. Szczepaniak LS, Nurenberg P, Leonard D, et al. Magnetic resonance spectroscopy to measure hepatic triglyceride content: prevalence of hepatic steatosis in the general population. *Am J Physiol Endocrinol Metab* 2005;288(2):E462-8. doi: 10.1152/ajpendo.00064.2004 [published Online First: 2004/09/02]
92. Clark JM, Brancati FL, Diehl AM. Nonalcoholic fatty liver disease. *Gastroenterology* 2002;122(6):1649-57. [published Online First: 2002/05/23]
93. Szczepaniak LS, Babcock EE, Schick F, et al. Measurement of intracellular triglyceride stores by H spectroscopy: validation in vivo. *Am J Physiol* 1999;276(5 Pt 1):E977-89. [published Online First: 1999/05/18]
94. Bohte AE, van Werven JR, Bipat S, et al. The diagnostic accuracy of US, CT, MRI and 1H-MRS for the evaluation of hepatic steatosis compared with liver biopsy: a meta-analysis. *Eur Radiol* 2011;21(1):87-97. doi: 10.1007/s00330-010-1905-5 [published Online First: 2010/08/04]
95. Banerjee R, Pavlides M, Tunnicliffe EM, et al. Multiparametric magnetic resonance for the non-invasive diagnosis of liver disease. *J Hepatol* 2014;60(1):69-77. doi: 10.1016/j.jhep.2013.09.002 [published Online First: 2013/09/17]
96. McPherson S, Jonsson JR, Cowin GJ, et al. Magnetic resonance imaging and spectroscopy accurately estimate the severity of steatosis provided the stage of fibrosis is considered. *J Hepatol* 2009;51(2):389-97. doi: 10.1016/j.jhep.2009.04.012 [published Online First: 2009/06/10]

For peer review only - http://bmjopen.bmj.com/site/about/guidelines.xhtml

97. Valls C, Iannacconne R, Alba E, et al. Fat in the liver: diagnosis and characterization. *Eur Radiol* 2006;16(10):2292-308. doi: 10.1007/s00330-006-0146-0 [published Online First: 2006/02/16]
98. van Werven JR, Hoogduin JM, Nederveen AJ, et al. Reproducibility of 3.0 Tesla magnetic resonance spectroscopy for measuring hepatic fat content. *J Magn Reson Imaging* 2009;30(2):444-8. doi: 10.1002/jmri.21837 [published Online First: 2009/07/25]
99. Longo R, Pollesello P, Ricci C, et al. Proton MR spectroscopy in quantitative in vivo determination of fat content in human liver steatosis. *J Magn Reson Imaging* 1995;5(3):281-5. [published Online First: 1995/05/01]
100. Longo R, Ricci C, Masutti F, et al. Fatty infiltration of the liver. Quantification by 1H localized magnetic resonance spectroscopy and comparison with computed tomography. *Invest Radiol* 1993;28(4):297-302. [published Online First: 1993/04/01]
101. Idilman IS, Aniktar H, Idilman R, et al. Hepatic steatosis: quantification by proton density fat fraction with MR imaging versus liver biopsy. *Radiology* 2013;267(3):767-75. doi: 10.1148/radiol.13121360 [published Online First: 2013/02/06]
102. Tang A, Tan J, Sun M, et al. Nonalcoholic fatty liver disease: MR imaging of liver proton density fat fraction to assess hepatic steatosis. *Radiology* 2013;267(2):422-31. doi: 10.1148/radiol.12120896 [published Online First: 2013/02/06]
103. Noureddin M, Lam J, Peterson MR, et al. Utility of magnetic resonance imaging versus histology for quantifying changes in liver fat in nonalcoholic fatty liver disease trials. *Hepatology* 2013;58(6):1930-40. doi: 10.1002/hep.26455 [published Online First: 2013/05/23]
104. Parkin E, O'Reilly DA, Plumb AA, et al. Digital histology quantification of intra-hepatic fat in patients undergoing liver resection. *Eur J Surg Oncol* 2015;41(8):1020-7. doi: 10.1016/j.ejso.2015.05.003 [published Online First: 2015/06/10]
105. Coe P, Williams S, Morris D, et al. Development of MR quantified pancreatic fat deposition as a cancer risk biomarker (PanORAMA) [submitted]. *Pancreatology* 2018

For peer review only

For peer review only - http://bmjopen.bmj.com/site/about/guidelines.xhtml

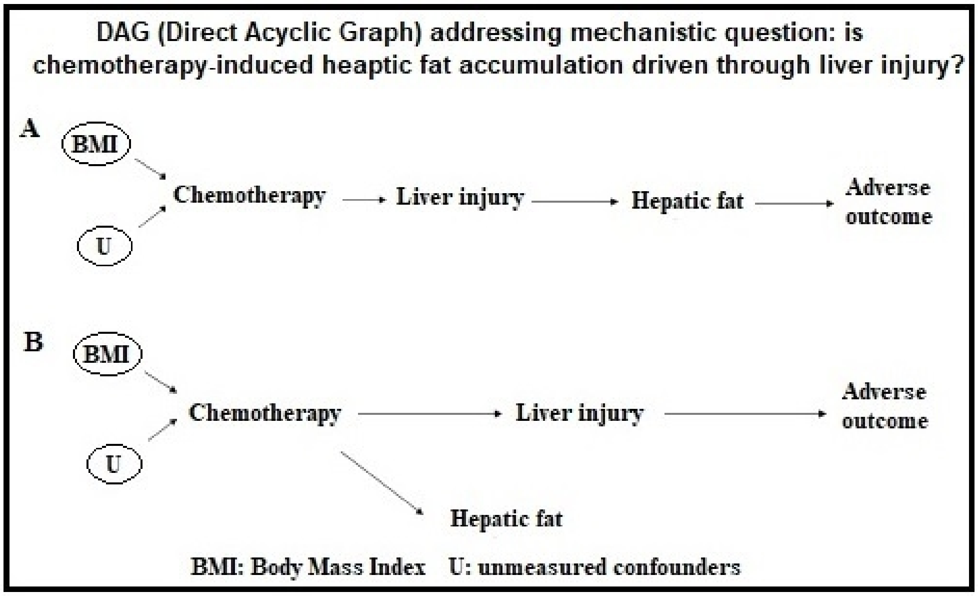

Direct Acyclic Graph

43x27mm (300 x 300 DPI)

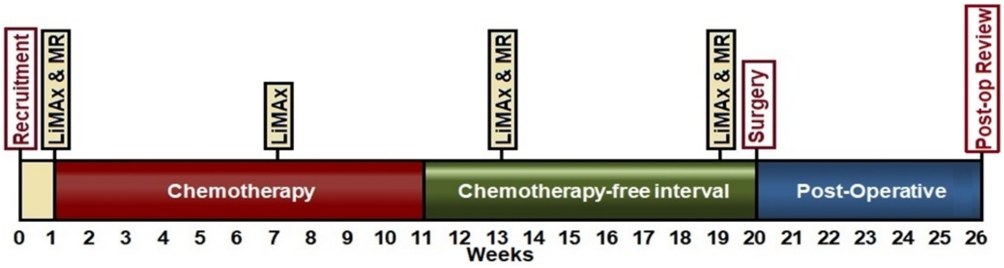

Study Participant Timeline

87x23mm (300 x 300 DPI)

APPENDIX 1 - STUDY SCHEDULE

				Standard care assessments		Experimental added assessments		
**Timeline (weeks)**		**Oncology Outpatient Department**	**Blood Tests**	**CT**	**Histology**	**LiMAx**	**MR**	**EQ-5D**
**BASELINE**		**Patient identification HPB MDT/ Oncology**		✓				
		**New Patient Oncology Clinic – CLiFF Study information**	✓					
**1**	Day 1	Oncology Clinic – Assessment, Bloods	✓			✓	✓	✓
	Day 2	**Chemotherapy- Cycle 1**						
**3**	Day 1	Oncology Clinic – Assessment, Bloods	✓					
	Day 2	**Chemotherapy- Cycle 2**						
**5**	Day 1	Oncology Clinic – Assessment, Bloods	✓					
	Day 2	**Chemotherapy- Cycle 3**						
**7**	Day 1	Oncology Clinic – Assessment, Bloods	✓			✓		
	Day 2	**Chemotherapy- Cycle 4**						
**9**	Day 1	Oncology Clinic – Assessment, Bloods	✓					
	Day 2	**Chemotherapy- Cycle 5**						
**11**	Day 1	Oncology Clinic – Assessment, Bloods	✓					
	Day 2	**Chemotherapy- Cycle 6**						
**13**	*Chemo finished	Oncology Clinic – Final Assessment, Bloods **ONCOLOGY DISCHARGE**	✓	✓		✓	✓	✓
**19**	**Post-Chemotherapy Week 8**							
	**Additional Visit to Christie Site** LiMAx and MR					✓	✓	✓
**Pre-Op** (~ Wk 19/20)		Pre-operative assessment clinic at Manchester Foundation Trust HPB Dept	✓					
**6 weeks post-op** (~ Wk 26)		Post-operative review clinic at Manchester Foundation Trust HPB Dept	✓		✓			✓

APPENDIX 2 – STUDY ASSESSMENTS

	Event	Baseline	Pre-chemo	2nd cycle	3rd cycle	4th cycle	5th cycle	6th cycle	Following last cycle of chemotherapy	Approx 8 wks post-chemo/ pre-op assessment	Post-op Assessment
**Standard Care Events/ Assessments**	History	✓									
	Physical examination	✓	✓	✓	✓	✓	✓	✓	✓	✓	✓
	Height	✓									
	Weight	✓	✓	✓	✓	✓	✓	✓	✓	✓	✓
	WHO perf score	✓	✓	✓	✓	✓	✓	✓	✓	✓	✓
	FBC	✓	✓	✓	✓	✓	✓	✓	✓	✓	✓
	Biochemistry [b]	✓	✓	✓	✓	✓	✓	✓	✓	✓	✓
	CEA	✓								✓	
	Coagulation [c]	✓	✓	✓	✓	✓	✓	✓	✓	✓	
	Pregnancy test [d]	✓									
	CT	✓							✓		
	Histopathology										✓
**Experimental Additional Events/ Assessments**	Informed Consent		✓								
	EQ-5D Score		✓						✓	✓	✓
	MR		✓						✓	✓	
	LiMAx		✓			✓			✓	✓	

For peer review only - http://bmjopen.bmj.com/site/about/guidelines.xhtml

BMJ Open

A prospective study of Change in Liver Function and Fat in patients with colorectal liver metastases undergoing pre-operative chemotherapy: protocol for the CLiFF study

Journal:	*BMJ Open*
Manuscript ID	bmjopen-2018-027630.R2
Article Type:	Protocol
Date Submitted by the Author:	12-Oct-2019
Complete List of Authors:	Parmar, Kat; University of Manchester, Division of Cancer Sciences, School of Medical Sciences, Faculty of Biology, Medicine and Health; Manchester Cancer Research Centre<br>O'Reilly, Derek; Central Manchester University Hospitals NHS Foundation Trust, Hepatobiliary Surgery<br>Valle, Juan; Christie NHS Foundation Trust, Oncology; University of Manchester, Division of Cancer Sciences, School of Medical Sciences, Faculty of Biology, Medicine and Health<br>Braun, Michael; Christie NHS Foundation Trust, Oncology<br>Naish, Jo; University of Manchester Institute of Cardiovascular Sciences<br>Williams, Steve; University of Manchester Centre for Imaging Sciences<br>Lloyd, William; University of Manchester Centre for Imaging Sciences<br>Malcomson, Lee; University of Manchester, Division of Cancer Sciences, School of Medical Sciences, Faculty of Biology, Medicine and Health; Christie NHS Foundation Trust<br>Cresswell, Katharine; Manchester University NHS Foundation Trust, Public Programmes Team, Research and Innovation Division<br>Bamford, Colin; NIHR Manchester Biomedical Research Centre, Cancer Patient and Public Advisory Group<br>Renehan, Andrew; University of Manchester, 1 Division of Cancer Sciences, School of Medical Sciences, Faculty of Biology, Medicine and Health; Christie NHS Foundation Trust
**Primary Subject Heading**:	Oncology
Secondary Subject Heading:	Surgery, Oncology, Radiology and imaging, Diagnostics
Keywords:	Colorectal surgery < SURGERY, Hepatobiliary surgery < SURGERY, CHEMOTHERAPY

SCHOLARONE™
Manuscripts

Type of article: study protocol (formatted for BMJ Open)

A prospective study of Change in Liver Function and Fat in patients with colorectal liver metastases undergoing pre-operative chemotherapy: protocol for the CLiFF study

Kat L Parmar,[1,2,3,4] Derek O'Reilly,[4] Juan W Valle,[1,3] Michael Braun,[3] Jo H Naish,[5] Steve R Williams,[6] William K Lloyd,[6] Lee Malcomson,[1,2,3] Katharine Cresswell,[2,7,8] Colin Bamford,[9] Andrew G Renehan[1,2,3]

[1] Division of Cancer Sciences, School of Medical Sciences, Faculty of Biology, Medicine and Health, University of Manchester

[2] Manchester Cancer Research Centre, NIHR Manchester Biomedical Research Centre, Manchester

[3] The Christie NHS Foundation Trust, Manchester

[4] Department of Hepatobiliary Surgery, Manchester University Foundation Hospitals, Manchester

[5] Division of Cardiovascular Sciences, School of Medical Science, Faculty of Biology, Medicine and Health, University of Manchester

[6] Centre for Imaging Sciences, School of Health Sciences, Faculty of Biology, Medicine and Health, University of Manchester

[7] Division of Evolution and Genomic Sciences, School of Biological Sciences, Faculty of Biology, Medicine and Health, University of Manchester

[8] Public Programmes Team, Research and Innovation Division, Manchester University NHS Foundation Trust, Manchester

[9] NIHR Manchester Biomedical Research Centre Cancer Patient and Public Advisory Group

Running title: *Liver fat and function after chemotherapy*

Keywords: liver injury, liver steatosis, liver resection, colorectal cancer metastases, chemotherapy

Correspondence to:

Miss Kat Parmar
Division of Cancer Sciences, School of Medical Sciences,
Faculty of Biology, Medicine and Health,
University of Manchester,
The Christie NHS Foundation Trust,
Wilmslow Road
Manchester,
M20 4BX UK
Tel: +44 161 3060870
E-mail: kat.parmar@manchester.ac.uk

Abstract: 266 words (max: 300); main text: 3818 words (max: 4000); x2 figures; 105 references supplemental material (2 pages); language: UK English.

For peer review only - http://bmjopen.bmj.com/site/about/guidelines.xhtml

ABSTRACT

Introduction

Pre-operative chemotherapy in patients undergoing resection for colorectal liver metastases (CLM) improves oncological outcomes. However, chemotherapy-associated liver injury (occurring in two patterns: vascular and fat deposition) is a real clinical concern prior to hepatic resection. After major liver resection, regeneration of the residual liver is a prerequisite for recovery and avoidance of liver failure, but this regenerative capacity may be hindered by chemotherapy. Thus, there is a need to predict for this serious complication. Over the past two decades, several tests and derived indices have been developed, which have failed to achieve clinical utility, mainly as they were indirect measurements of liver function. Here, we will use a novel test of liver function (the LiMAx test), and measure liver fat using magnetic resonance (MR) imaging.

Methods and analysis

This prospective study will assess changes in liver function longitudinally, measured by the LiMAx test, and liver fat, measured by advanced MR imaging using both MR spectroscopy and the modified Dixon method, in up to 35 patients undergoing pre-operative chemotherapy for CLM. The primary outcomes will be the changes in liver function and fat compared to baseline pre-chemotherapy measurements. Secondary outcome measures include: routinely measured liver function blood tests, anthropometric measurements, post-operative histology and digital quantification of fat, post-operative complications and mortality and quality of life.

Ethics and dissemination

The study was approved by a National Health Service Research Ethics Committee and registered with the Health Research Authority. Dissemination will be via international and national conferences, and the NIHR network. Manuscripts will be published.

Trial Registration Number:

This study is registered online at www.clinicaltrials.gov (registration number NCT03562234).

For peer review only - http://bmjopen.bmj.com/site/about/guidelines.xhtml

Strengths and limitations of this study

- This is an evaluation of the effects of pre-operative chemotherapy upon liver function and fat using novel techniques, addressing the important clinical issue of liver injury prior to hepatic resection
- These non-invasive techniques permit repeated measurements to assess change in liver function and fat over time, permitting interpretation of causality – this has not been possible in the past
- Chemotherapy-related changes in liver fat and function will be assessed for reversibility and correlated with both standard assessment measures and post-operative histology, providing insight into the scale of the injuries prior to the post-chemotherapy recovery period
- Changes and recovery in liver fat and function will be correlated with post-operative morbidity and mortality
- A potential limitation is that this is a single-site observational study with a relatively small sample size

For peer review only

For peer review only - http://bmjopen.bmj.com/site/about/guidelines.xhtml

BACKGROUND

Colorectal cancer (CRC) is the second most common cause of cancer death in the UK.[1] Death is usually due to metastatic disease, with the most common site being the liver (colorectal liver metastases - CLM). There is an incidence of 15-20% CLM at initial diagnosis of CRC[2] with up to 50% of patients ultimately developing CLM.[3-5 6]

Surgical resection of CLM is the only single-modality therapy associated with cure and remains the mainstay of treatment.[7] The resectability of CLM depends upon the volume and function of the future liver remnant (FLR). Volume alone is not an ideal assessment of liver functionality as it cannot reliably predict outcome.[7-11 12] FLR function is decreased in patients with parenchymal disease, despite being of equal volume to patients with heathy livers,[13-15] and liver disease may result in impaired liver regeneration.[7 11 16-18] In the presence of impaired liver function the surgical strategy may need to be adapted, either to increase the volume of the FLR or perform a less extensive resection.[2 19] Accurate pre-operative assessment of liver function is therefore crucial.[20]

With best supportive care, the median survival of patients with CLMs is 6-13 months (where five-year survival is rare).[21] In patients fit enough to receive combined hepatic resection and chemotherapy, the 5-year survival improves to 50%.[22 23 24 24-27] Pre-operative chemotherapy is used in the setting of both resectable and unresectable CLM, with the intentions of (i) improving survival (neoadjuvant approach) in operable CLM; and (ii) converting inoperable CLM into operable (downsizing approach). Approximately 70% of patients presenting with CLM are initially deemed unresectable,[28] and after systemic chemotherapy, up to 40% of patients may be judged resectable.[29 30-34] Outcomes in these "downsized" cases can be similar to those initially presenting with resectable disease.[35 36] In the setting of operable CLM, the randomized EPOC trial reported a 7.3% improvement in 3-year progression-free survival with addition of chemotherapy versus resection alone for CLM, and highlighted the

For peer review only - http://bmjopen.bmj.com/site/about/guidelines.xhtml

issue that organ-specific resectional surgery does not address the systemic process, leading to high post-operative recurrence rates.[37]

The main concern regarding the pre-operative administration of chemotherapy for CLM is direct pathologic changes to the liver, known as chemotherapy-associated liver injury (CALI). CALIs comprise two main types of liver injury: vascular changes and fatty changes. These injuries are not mutually exclusive; patients who have developed fatty changes can simultaneously develop vascular changes.[7] There are many inconsistent reports on the types of liver injury seen and their associations with specific chemotherapy regimens.[11 30 37-58] There is considerable heterogeneity in the methods used to assess changes to the liver parenchyma. Earlier studies used CT scans and liver biopsies[38-41] and later studies used post-operative histology, reported using a variety of scoring systems.[11 30 37 42-58] Radiation exposure resulting from CT scans and the invasive nature of liver biopsies mean that neither technique is suited to providing repeated measurements, and post-operative specimens provide only a one-off assessment of the liver parenchyma. None of these assessment tools are therefore ideally suited to measuring change over time.

The link between CALIs and post-operative morbidity and mortality remains unclear with reports to date being mixed.[30 37 42-45 47-53 55-59] Some studies such as the one by Karoui et al[47] have demonstrated that CALIs increased the risk of post-operative liver failure (11% v 0%), with others such as Vauthey et al[48] demonstrating increased post-operative mortality (increased 90-day mortality in patients with steatohepatitis 14.7% v 1.6%, $P=0.001$; steatohepatitis associated with pre-operative irinotecan 20.2% v 4.4%, $P<0.001$).

Post-operative morbidity and mortality are often related to inadequate function of the remnant liver, resulting in post-operative liver failure - an important cause of mortality after partial liver resection.[14 60 61] A meta-analysis estimated the overall incidence of liver failure after hepatectomy to lie between 0.7% and 9.1%,[34] with most recent studies reporting liver

For peer review only - http://bmjopen.bmj.com/site/about/guidelines.xhtml

failure rates within this range.[62 58] The improved detection of CALIs, assessment of their impact upon post-operative outcomes and pre-operative assessment of the FLR are thus important clinical issues. Several tests and derived indices are in use, including conventional blood parameters of liver function (liver enzymes, albumin, bilirubin, INR), Child-Pugh Classification, scintigraphy, galactose elimination test and indocyanine green (ICG) clearance rate. These assessments are suboptimal as they comprise indirect measurements of liver function.

Advances in chemotherapy and hepatic surgery have expanded the pool of candidates for potentially curative hepatic resection for CLM,[7] many of whom will undergo pre-operative chemotherapy. Recognition of CALIs emphasises the need to fully assess each patient and utilise individualised planning, giving attention to the planned extent of resection, the choice of chemotherapy regimen and the potential consequences upon the hepatic parenchyma. Future challenges include the refinement of liver function assessment and the establishment of better methods for evaluation and diagnosis of post-chemotherapy liver injury.[7]

This study aims to address these challenges by utilising the following two novel techniques for assessing liver function and liver fat, measuring these repeatedly before after chemotherapy, and correlating these changes to post-operative outcomes. Performing repeated measurements over time of both liver fat deposition and liver function during and after the cessation of chemotherapy will permit better interpretation of causality and potential reversibility. This will address the mechanistic question shown in **Figure 1**: is chemotherapy-induced hepatic fat accumulation driven through liver injury as shown in Hypothesis A, or unrelated as in Hypothesis B?

For peer review only

Figure 1 (See separate image file)

Understanding these relationships and making use of the observation that pre-operative chemotherapy is a model of an extreme accelerated phenotype of liver fat deposition may also help to address some of the underlying mechanistic questions in obesity-related cancers.

The LiMAx test

The Liver Maximum capacity (LiMAx) test was developed as a novel clinical evaluation of liver function, aimed at overcoming the difficulties in accurately assessing pre-operative liver function prior to hepatic resection.[63] It is based on the metabolism of a ^{13}C-labelled substrate by the hepatocyte-specific enzyme system P450 CYP1A2, the activity of which is not influenced by drugs or genetic variations,[64] is distributed ubiquitously throughout the liver,[65] and shows a clear discrimination between normal and abnormal liver function independent of cholestasis.[66] The intravenous administration of ^{13}C-methacetin results in a significant alteration of the normal expired breath $^{13}CO_2$:$^{12}CO_2$ ratio[67] detected by breath analysis performed by an infrared spectroscopy-based FLIP device.

The LiMAx test was initially used in pre- and post-operative hepatectomy assessment, where resection of a specific percentage of functional liver volume was found to lead to an equivalent reduction in LiMAx value.[68] The LiMAx test was found to represent an accurate surrogate of liver function capacity,[68] to be an accurate predictor of post-operative liver failure and mortality,[68 63 69 70] and to be unaffected by age and gender.[71]

The LiMAx test has been evaluated as a tool with which to diagnose CALIs. LiMAx was found to be superior to other methods of assessing liver function both in detecting CALIs and their resolution, when compared to standard biochemical liver parameters (INR, bilirubin,

For peer review only - http://bmjopen.bmj.com/site/about/guidelines.xhtml

aspartate transaminase) and ICG-clearance rates.[72 73] However, it has never been used repeatedly in the setting of CLM or correlated with post-operative outcomes.

Advanced MR imaging

Traditional assessment methods failed to provide an ideal measurement tool for liver fat content (hepatic fat fraction – HFF). Ultrasonography scan (USS) and computed tomography (CT) have the ability to demonstrate fatty infiltration, however, they are unable to quantify HFF.[74-77] USS is preferred for qualitative assessment because CT sensitivity is time-dependent and protocol specific[78] and is not suitable for use in longitudinal monitoring due to its reliance on ionising radiation.[79] On the other hand, USS is low-cost, safe, and readily accessible.[79] However, USS has limited repeatability and reproducibility, and the outputs have only modest correlation with biopsy results.[78 80] Liver biopsy provides a quantitative method for the assessment of fatty liver disease, however, this is an invasive procedure hampered by sampling errors as fatty infiltrations can be unevenly distributed throughout the liver,[81-83] significant inter-observer variability[84] and potential complications such as bleeding and mortality.[85]

Magnetic resonance (MR) imaging using standard pulse sequences is insensitive to diffuse fatty infiltrations.[86-88] Techniques have been developed with the aim of further exploiting the differences in resonance frequencies between water and fat proton signals in order to assess HFF with increased accuracy, including magnetic resonance spectroscopy (MRS)[89] and the modified Dixon technique (mDixon).[90]

MRS utilises a much larger sample of the liver as compared to a typical biopsy sample, minimising the chance of sampling error, and is sensitive enough to detect small amounts of fat that may be histologically undetectable.[91] It is considered to be the optimal non-invasive method for the accurate assessment of liver fat,[92] being the technique shown to produce the most reliable and reproducible measurements.[79 81 91 93-97 98] MRS has been validated as an

For peer review only - http://bmjopen.bmj.com/site/about/guidelines.xhtml

accurate technique against the histological evaluation of fatty liver changes in multiple studies.[81 93-96 99 100] However, MRS has not achieved widespread clinical use despite the undisputed strengths of this technique. This may be due to poor availability of expertise and equipment outside the research setting.[90]

Techniques of quantitative magnetic resonance imaging have recently been introduced for the acquisition of proton density fat fraction maps covering large portions of the liver. These modified Dixon techniques (mDixon) show promising results in assessing HFF in comparisons with histological scoring systems[101-103] and correlate well with MRS.[90]

The Manchester Obesity and Cancer Research Group run a long-term research programme developing non-invasive imaging techniques to quantify liver fat. We have previously validated the use of MRS and the mDixon method against digital histological determination of fatty infiltration in post-surgical specimens, quantifying and characterising fat deposition in patients with CLM undergoing resection.[104 105] These previous studies were cross-sectional whereas the current study will evaluate changes over time in order to better assess their relationship to chemotherapy and their potential reversibility.

For peer review only

The proposed MR imaging protocols in the current study will be as follows:

MRS

Single volume 1H spectra are acquired using Stimulated Echo Acquisition Mode (STEAM) spectroscopy (TR = 2000 ms, TE = 10 ms, FA = 90°. 16 dynamic scans are acquired without averaging for a 15 mm3 voxel of interest (VOI) with a 32 s duration. This is repeated three times with VOIs positioned to avoid CLM and partial volume averaging with large blood vessels and bile ducts.

mDixon

For peer review only - http://bmjopen.bmj.com/site/about/guidelines.xhtml

A six-echo 3D gradient echo sequence is used, with first TE at 0.92 ms and a delta of 0.7 ms (TR = 5.3 ms, FA = 5°). Full liver coverage is acquired with 67 axial slices and 3 mm2 in-plane and 6 mm through plane resolution, interpolated to 2 mm2 in-plane and 3 mm through plane. Parallel acquisition used with a SENSE factor of 1.5 in the AP plane. The full volume is acquired in a single breath hold for a duration of 12 seconds. Quantitative fat fraction maps are calculated using a dedicated scanner software package. A representative fat fraction for each subject is calculated using the average from three liver-tissue regions of interest positioned on a central slice, using the same criteria as for the spectroscopy.

AIMS

To prospectively assess the changes in liver function and fat resulting from the administration of pre-operative chemotherapy prior to hepatic resection for colorectal liver metastases (Change in Liver Function and Fat – the CLiFF study). These changes will be assessed repeatedly using the novel techniques of the LiMAx test and advanced MR imaging as demonstrated in **Figure 2**, then correlated with standard assessments and post-operative outcomes. To our knowledge, there is no prospective evaluation of this type to-date.

Figure 2 – Study Participant Timeline (See separate image file)

Primary outcome measures

1. Change in liver function measured by the LiMAx test performed using the FLIP device, comparing baseline measurements with measurements during and after chemotherapy.
2. Change in liver fat as a percentage (Hepatic Fat Fraction – HFF) measured by advanced Magnetic Resonance imaging (utilising both Magnetic Resonance Spectroscopy and the modified Dixon method), comparing baseline measurements with measurements during and after chemotherapy.

Secondary outcome measures

For peer review only - http://bmjopen.bmj.com/site/about/guidelines.xhtml

1) Changes in routinely collected liver enzymes by blood testing
2) Digital histological quantification of intrahepatic fat
3) Correlations with BMI and image-determined anthropometric measurements e.g. subcutaneous adipose tissue (SAT), visceral adipose tissue volumes (VAT) and muscle mass
4) Grade of steatosis scores (NAS-CRN) from routine histology reporting
5) Post-operative outcomes, including peri-operative mortality and morbidity (Clavien-Dindo classification, Comprehensive Complication Index, International Study Group for Liver Surgery complications).
6) Change in health-related quality of life by measurement of EQ-5D score

METHODS

Study design

This is a non-randomised prospective observational study of up to 35 patients with CLM.

Study setting

The study is sponsored by the University of Manchester and will be performed at The Christie NHS Foundation Trust and the University of Manchester Wolfson Molecular Imaging Centre. Participant identification will take place at the joint Christie-Manchester Foundation Trust hepatopancreatobiliary (HPB) multidisciplinary team (MDT) meeting. Team members will screen all patients for whom an MDT decision is made that a course of pre-operative chemotherapy is indicated prior to potentially curative hepatic resection of CLM.

Institutions

From October 6, 2014, a single Improving Outcomes Guidance-compliant HPB Service has been located at the Manchester Royal Infirmary site at Manchester University NHS Foundation Trust. It hosts the regional specialist HPB MDT meeting. All patients selected to

For peer review only

undergo neoadjuvant chemotherapy prior to hepatic resection from this MDT will receive this treatment via the oncology service delivered from the Christie Hospital.

The Wolfson Molecular Imaging Centre is part of the University of Manchester. This centre provides state-of-the art imaging facilities and radiological expertise in the advanced imaging techniques of MRS and mDixon. The Philips 1.5T scanner at WMIC was upgraded to provide mDixon in addition to the established MRS.

Investigators

The CLiFF study is led by a dedicated clinical research fellow, as part of a University-registered thesis. The supervisory team provide the relevant expertise in surgical cancer research, hepatobiliary surgery, medical oncology and imaging science, with additional research and statistical support available as required.

Inclusion criteria

Patients with liver metastases of colorectal origin, undergoing pre-operative standard chemotherapy, with the following criteria:

1. Histologically verified adenocarcinoma of the colon or rectum with radiological evidence of potentially resectable liver metastases;
2. No evidence of unresectable non-hepatic metastatic disease;
3. Adequate haematological and hepatic function defined by: Hb $\geq$ 10g/dl, WBC $\geq$ 3.0 x 10^9/L, ANC $\geq$ 1.5 x 10^9/L, platelet count $\geq$ 100,000/ mm^3, total bilirubin < 30 μmol/L, serum AST, ALT and alkaline phosphatase $\leq$ 5 x upper limit of normal;
4. Written informed consent and able to attend long-term follow-up.

Exclusion criteria

For peer review only - http://bmjopen.bmj.com/site/about/guidelines.xhtml

1. Presence of a medical or psychiatric condition that impairs their ability to give informed consent
2. Presence of any other serious uncontrolled medical conditions
3. Evidence of unresectable non-hepatic metastatic disease

Patient identification

Patients will be screened for inclusion initially via discussion at the specialist HPB MDT meeting and by referral to the Medical Oncology service. Subsequent to an MDT decision that pre-operative chemotherapy is indicated prior to potentially curative resection of colorectal liver metastases, the CLiFF study team will screen for the remaining inclusion criteria. Potential participants will be invited to join the study via the provision of verbal and written information regarding the CLiFF study. Confirmation of the wish to participate will subsequently be confirmed and informed consent taken prior to beginning chemotherapy.

Data collection

Participants will undergo assessment of liver function by LiMAx testing and HFF by advanced MR imaging at the times outlined in the study schedule shown in Appendix 1 and 2. All clinicians involved in the medical and surgical treatment of the study participants will be blinded to the results of the LiMAx testing and MR imaging. The study MR images will not be reported by a clinical radiologist. All other aspects of treatment will remain unchanged and proceed as per standard care.

Data collection will include LiMAx tests and MR results, in addition to the results from all other assessments outlined in Appendix 2. The LiMAx tests and MR scans will be performed directly by the clinical research fellow. The remaining information will be taken from the medical notes, comprising the results from routine oncology outpatient assessments, the post-operative histology report and the post-operative outcomes following hepatic resection.

For peer review only - http://bmjopen.bmj.com/site/about/guidelines.xhtml

Informed consent will be taken for the research team to access this information from the medical records.

Data management

The NHS code of confidentiality will be observed, with only the clinical care team and team directly involved in the research having access to any identifiable data. All data will be pseudonymised with a unique identifier and stored in a secure encrypted database. A key to patient identifiers will be stored in a separate encrypted document. Data stored by the research team will therefore not contain patient identifiers. Data will be collected and stored in accordance with the General Data Protection Regulation (GDPR) and Data Protection Act 2018. The University of Manchester, as Data Controller for this study, takes responsibility for the protection of any personal information collected by this study. All researchers will be appropriately trained in data protection.

Statistical analysis & power calculation

Our previous work reported a mean (±sd) Hepatic Fat Fraction of 4 (±2) %.[105] Following chemotherapy, we estimate at least a 50% increase in HFF i.e. increase to mean (sd) = 6 ± 2.5%. We have planned a recruitment of at least 35 patients. As attrition (for multiple reasons) is common in this type of study, of the 35 patients, we conservatively estimate that 25 patients will have complete data from at least two MR evaluations. The resultant power is 98% (alpha: 0.05; within-person correlation: 0.7).

For the LiMAx testing, which is more frequent in the study schedule, we estimate at least 32 patients (10% attrition rate) will have complete data from two LiMAx tests. Based on the Lock et al [2017] study in patients undergoing pre-resection chemotherapy[73] [mean (±sd): 340 ± 95 vs. 391 ± 82 µg/kg/h], the resultant power is 84% (alpha: 0.05; within-person correlation: 0.7).

For peer review only - http://bmjopen.bmj.com/site/about/guidelines.xhtml

Statistical support will be provided where required by the University of Manchester Cancer Data Science Team. The main analysis will focus on intra-patient change in both liver function (measured by the LiMAx test) and hepatic fat fraction (measured by advanced MR imaging) which occurs subsequent to chemotherapy, and their recovery prior to surgery. These changes will be related to subsequent clinical course, CT volumetry and histopathological analysis of the resected liver. We will explore the use of ANOVA methods and mixed-effects methods to account for within-person correlations. For each time point, standard approaches to categorical (chi-squared test) and continuous (Mann-Whitney U test) variables will be used. Correlation matrices will be constructed to assess relationships between LiMAx and HFF with other continuous variables using Spearman's Rank Correlation Coefficient.

To account for multiple testing, a p-value of <0.01 will be considered to indicate statistical significance. Predictors of HFF at each time point will be explored using multivariable linear regression models. To reduce the anticipated right skewness of the distributions, logarithmic transformation will be explored.

Because of the large number of variables and the anticipated high levels of correlations, we will use factor-cluster methods. There will be at least five clusters: patient-related factors; routine blood measures; CT-derived anthropometrics; LiMAx readouts; and MR readouts. Separate models will be developed for each cluster and significant ($p <0.05$) variables selected for the final model.

Anticipated recruitment

At the specialist HPB MDT, there are approximately 120 resections per year performed for CLM. Approximately half these patients will receive pre-operative chemotherapy. Of these 60 patients, 40 will have their chemotherapy delivered at the oncology centre and will be potentially eligible for recruitment. Over the past 4 years, the numbers of liver resections

For peer review only - http://bmjopen.bmj.com/site/about/guidelines.xhtml

For peer review only

performed for CLM has consistently increased year on year. We therefore anticipate that the recruitment target of 35 patients to be recruited and followed up within two years is achievable.

Quality assurance

The quality of this study has been assessed by the following means:

- Departmental review within the University of Manchester
- Peer review by professionals with relevant expertise (Clinical trialists, statisticians, surgeons, oncologists and imaging scientists)
- Review by the Research Practice Governance Team at the University of Manchester (Sponsor Institution) and the Research & Development team at the Christie NHS Foundation Trust (host)
- Peer review arranged by the North West Innovation Service as part of a successful competitive funding application
- Review by the Royal College of Surgeons research department as part of a successful competitive application for a research fellowship
- Independent peer review as part of the registration process for ClinicalTrials.gov, a publicly accessible database of worldwide research studies, maintained by the National Library of Medicine at the National Institutes for Health (US) (NCT03562234)

Study Timetable

The anticipated study timetable is outlined in Table 1.

Table 1: Study Timetable

Time Period	Date
Study Set-Up and Approvals	01/04/2018 - 01/09/2018
Participant Recruitment	01/10/2018 - 01/04/2020
Data collection	01/04/2020- 01/10/2020

For peer review only - http://bmjopen.bmj.com/site/about/guidelines.xhtml

Data Analysis and Write-Up	01/10/2020 – 31/03/2021

ETHICS AND DISSEMINATION

Ethical approval

Ethical approval for this study was granted by the National Health Service North West Research Ethics Committee following the meeting held on 27th July 2018 (REC Reference 18/NW/0531).

Registration

This study was registered, reviewed and approved by the Health Research Authority (HRA). It has been registered with the sponsor institution (University of Manchester) and approved by their Research Practice Governance Team. The host institution (Christie NHS Foundation Trust) Research & Development team have issued approval of their capability and capacity to host this study. The study was registered online at www.clinicaltrials.gov, registration number NCT03562234.

Patient and Public Involvement

The Public Programmes Team at the Manchester University NHS Foundation Trust hosted a discussion group for the CLiFF Study with six members of the NIHR Manchester Biomedical Research Centre funded Cancer Research Advisory Panel on 24/05/2018. The six attending members were all patients/carers with personal experience of CLM treatment. This event was attended by the Principle Investigator to actively seek and hear the views of the patient and public representatives. The group unanimously found that this area of research was important, found no feasibility or acceptability issues with the study and thought it likely that they would take part if asked. The following modifications were made in response to the session:

1) **Amendments to Participant Information Sheet:**

For peer review only - http://bmjopen.bmj.com/site/about/guidelines.xhtml

All group suggestions were incorporated, including the requested addition of a clearly highlighted opening statement explaining that treatment would not be modified as the result of participating in this study.

2) Training to use long-term intravenous access devices:

Patients who had previously undergone pre-operative chemotherapy for CLM expressed frustration that whilst many of them had long-term intravenous access devices for the duration of their treatment, inadequate training to access these devices resulted in additional exposure to needles. They requested that where possible, these long-term devices be used for the intravenous injection component of the LiMAx test. In response to this request, the Principal Investigator underwent additional training to ensure that long-term intravenous access devices could be utilised for the LiMAx tests performed in this study.

One patient member of the Cancer Research Advisory Panel contributed substantially and subsequently became a co-author of this protocol paper (CB).

Dissemination

This study will be submitted for presentation at national or international surgical conferences. Manuscript(s) will be prepared following close of the study.

Figure Legends

Figure 1: Direct Acyclic Graph

Figure 2: Study Participant Timeline

Authorship contributions

The original study concept was conceived by JWV, DOR and AGR. The study design was developed by KLP, MB, AGR, DOR and CB. The imaging protocols were developed by

For peer review only - http://bmjopen.bmj.com/site/about/guidelines.xhtml

SRW, JHN and WKL. Study set up was carried out by KLP, LM, AGR, DOR , KC, MB, JHN, SRW and WKL. The present protocol was written by DOR, AGR and KLP. All authors (KLP, DOR, JWV, MB, JHN, SRW, WKL, LM, KC, CB, AGR) contributed to the revision and preparation of this manuscript.

This research has received funding from the following sources:

Humedics GMBH; National Institute for Health Research Manchester Biomedical Research Centre; North West NHS Innovation service; The Christie NHS Foundation Trust charitable funds; The Royal College of Surgeons of England Fellowship scheme

Conflicts of interest: AGR reports personal fees from Merck Serona, personal fees from Janssen-Cilag, grants and personal fees from Sanofi Pasteur MSD, outside the submitted work. The remaining authors declare no conflicts.

For peer review only

References

1. CRUK. Cancer Research UK - Bowel Cancer Statistics 2018 [Available from: http://www.cancerresearchuk.org/health-professional/cancer-statistics/statistics-by-cancer-type/bowel-cancer accessed 2/04/2018.
2. Fiorentini G, Sarti D, Aliberti C, et al. Multidisciplinary approach of colorectal cancer liver metastases. *World J Clin Oncol* 2017;8(3):190-202. doi: 10.5306/wjco.v8.i3.190 [published Online First: 2017/06/24]
3. Manfredi S, Lepage C, Hatem C, et al. Epidemiology and management of liver metastases from colorectal cancer. *Ann Surg* 2006;244(2):254-9. doi: 10.1097/01.sla.0000217629.94941.cf [published Online First: 2006/07/22]
4. Nordlinger B, Van Cutsem E, Gruenberger T, et al. Combination of surgery and chemotherapy and the role of targeted agents in the treatment of patients with colorectal liver metastases: recommendations from an expert panel. *Ann Oncol* 2009;20(6):985-92. doi: 10.1093/annonc/mdn735 [published Online First: 2009/01/21]
5. Adam R, De Gramont A, Figueras J, et al. The oncosurgery approach to managing liver metastases from colorectal cancer: a multidisciplinary international consensus. *Oncologist* 2012;17(10):1225-39. doi: 10.1634/theoncologist.2012-0121 [published Online First: 2012/09/11]
6. Renehan AG, Egger M, Saunders MP, et al. Impact on survival of intensive follow up after curative resection for colorectal cancer: systematic review and meta-analysis of randomised trials. *BMJ* 2002;324(7341):813. [published Online First: 2002/04/06]
7. Zorzi D, Laurent A, Pawlik TM, et al. Chemotherapy-associated hepatotoxicity and surgery for colorectal liver metastases. *Br J Surg* 2007;94(3):274-86. doi: 10.1002/bjs.5719 [published Online First: 2007/02/23]
8. Schroeder RA, Marroquin CE, Bute BP, et al. Predictive indices of morbidity and mortality after liver resection. *Ann Surg* 2006;243(3):373-9. doi: 10.1097/01.sla.0000201483.95911.08 [published Online First: 2006/02/24]
9. Dinant S, de Graaf W, Verwer BJ, et al. Risk assessment of posthepatectomy liver failure using hepatobiliary scintigraphy and CT volumetry. *J Nucl Med* 2007;48(5):685-92. doi: 10.2967/jnumed.106.038430 [published Online First: 2007/05/04]
10. Erdogan D, Heijnen BH, Bennink RJ, et al. Preoperative assessment of liver function: a comparison of 99mTc-Mebrofenin scintigraphy with indocyanine green clearance test. *Liver Int* 2004;24(2):117-23. doi: 10.1111/j.1478-3231.2004.0901.x [published Online First: 2004/04/14]
11. Fernandez FG, Ritter J, Goodwin JW, et al. Effect of steatohepatitis associated with irinotecan or oxaliplatin pretreatment on resectability of hepatic colorectal metastases. *J Am Coll Surg* 2005;200(6):845-53. doi: 10.1016/j.jamcollsurg.2005.01.024 [published Online First: 2005/06/01]
12. Schindl MJ, Redhead DN, Fearon KC, et al. The value of residual liver volume as a predictor of hepatic dysfunction and infection after major liver resection. *Gut* 2005;54(2):289-96. doi: 10.1136/gut.2004.046524 [published Online First: 2005/01/14]
13. Yigitler C, Farges O, Kianmanesh R, et al. The small remnant liver after major liver resection: how common and how relevant? *Liver Transpl* 2003;9(9):S18-25. doi: 10.1053/jlts.2003.50194 [published Online First: 2003/08/28]
14. Farges O, Belghiti J, Kianmanesh R, et al. Portal vein embolization before right hepatectomy: prospective clinical trial. *Ann Surg* 2003;237(2):208-17. doi: 10.1097/01.SLA.0000048447.16651.7B [published Online First: 2003/02/01]
15. Wakabayashi H, Ishimura K, Okano K, et al. Application of preoperative portal vein embolization before major hepatic resection in patients with normal or abnormal liver parenchyma. *Surgery* 2002;131(1):26-33. [published Online First: 2002/01/29]

For peer review only - http://bmjopen.bmj.com/site/about/guidelines.xhtml

16. Folprecht G, Gruenberger T, Bechstein WO, et al. Tumour response and secondary resectability of colorectal liver metastases following neoadjuvant chemotherapy with cetuximab: the CELIM randomised phase 2 trial. *Lancet Oncol* 2010;11(1):38-47. doi: 10.1016/S1470-2045(09)70330-4 [published Online First: 2009/11/28]
17. Fausto N, Campbell JS, Riehle KJ. Liver regeneration. *Hepatology* 2006;43(2 Suppl 1):S45-53. doi: 10.1002/hep.20969 [published Online First: 2006/02/01]
18. Ikeda Y, Matsumata T, Takenaka K, et al. Preliminary report of tumor metastasis during liver regeneration after hepatic resection in rats. *Eur J Surg Oncol* 1995;21(2):188-90. [published Online First: 1995/04/01]
19. Nagashima I, Takada T, Adachi M, et al. Proposal of criteria to select candidates with colorectal liver metastases for hepatic resection: comparison of our scoring system to the positive number of risk factors. *World J Gastroenterol* 2006;12(39):6305-9. [published Online First: 2006/10/31]
20. Schneider PD. Preoperative assessment of liver function. *Surg Clin North Am* 2004;84(2):355-73. doi: 10.1016/S0039-6109(03)00224-X [published Online First: 2004/04/06]
21. Khan AZ, Toogood GJ. The case for adjuvant chemotherapy. Against. *Ann R Coll Surg Engl* 2008;90(6):454-5. [published Online First: 2008/09/09]
22. Morris EJ, Forman D, Thomas JD, et al. Surgical management and outcomes of colorectal cancer liver metastases. *Br J Surg* 2010;97(7):1110-8. doi: 10.1002/bjs.7032 [published Online First: 2010/07/16]
23. Mattar RE, Al-Alem F, Simoneau E, et al. Preoperative selection of patients with colorectal cancer liver metastasis for hepatic resection. *World J Gastroenterol* 2016;22(2):567-81. doi: 10.3748/wjg.v22.i2.567 [published Online First: 2016/01/27]
24. Abdalla EK, Vauthey JN, Ellis LM, et al. Recurrence and outcomes following hepatic resection, radiofrequency ablation, and combined resection/ablation for colorectal liver metastases. *Ann Surg* 2004;239(6):818-25; discussion 25-7. [published Online First: 2004/05/29]
25. Choti MA, Sitzmann JV, Tiburi MF, et al. Trends in long-term survival following liver resection for hepatic colorectal metastases. *Ann Surg* 2002;235(6):759-66. [published Online First: 2002/05/30]
26. Fernandez FG, Drebin JA, Linehan DC, et al. Five-year survival after resection of hepatic metastases from colorectal cancer in patients screened by positron emission tomography with F-18 fluorodeoxyglucose (FDG-PET). *Ann Surg* 2004;240(3):438-47; discussion 47-50. [published Online First: 2004/08/21]
27. Pawlik TM, Scoggins CR, Zorzi D, et al. Effect of surgical margin status on survival and site of recurrence after hepatic resection for colorectal metastases. *Ann Surg* 2005;241(5):715-22, discussion 22-4. [published Online First: 2005/04/26]
28. Simmonds PC, Primrose JN, Colquitt JL, et al. Surgical resection of hepatic metastases from colorectal cancer: a systematic review of published studies. *Br J Cancer* 2006;94(7):982-99. doi: 10.1038/sj.bjc.6603033 [published Online First: 2006/03/16]
29. Nordlinger B, Van Cutsem E, Rougier P, et al. Does chemotherapy prior to liver resection increase the potential for cure in patients with metastatic colorectal cancer? A report from the European Colorectal Metastases Treatment Group. *Eur J Cancer* 2007;43(14):2037-45. doi: 10.1016/j.ejca.2007.07.017 [published Online First: 2007/09/04]
30. Adam R, Delvart V, Pascal G, et al. Rescue surgery for unresectable colorectal liver metastases downstaged by chemotherapy: a model to predict long-term survival. *Ann Surg* 2004;240(4):644-57; discussion 57-8. [published Online First: 2004/09/24]
31. Adam R, Pascal G, Castaing D, et al. Tumor progression while on chemotherapy: a contraindication to liver resection for multiple colorectal metastases? *Ann Surg* 2004;240(6):1052-61; discussion 61-4. [published Online First: 2004/12/01]
32. Abdalla EK, Barnett CC, Doherty D, et al. Extended hepatectomy in patients with hepatobiliary malignancies with and without preoperative portal vein embolization. *Arch Surg* 2002;137(6):675-80; discussion 80-1. [published Online First: 2002/06/07]

For peer review only - http://bmjopen.bmj.com/site/about/guidelines.xhtml

33. Jaeck D, Oussoultzoglou E, Rosso E, et al. A two-stage hepatectomy procedure combined with portal vein embolization to achieve curative resection for initially unresectable multiple and bilobar colorectal liver metastases. *Ann Surg* 2004;240(6):1037-49; discussion 49-51. [published Online First: 2004/12/01]
34. van den Broek MA, Olde Damink SW, Dejong CH, et al. Liver failure after partial hepatic resection: definition, pathophysiology, risk factors and treatment. *Liver Int* 2008;28(6):767-80. doi: 10.1111/j.1478-3231.2008.01777.x [published Online First: 2008/07/24]
35. Adam R, Avisar E, Ariche A, et al. Five-year survival following hepatic resection after neoadjuvant therapy for nonresectable colorectal. *Ann Surg Oncol* 2001;8(4):347-53. [published Online First: 2001/05/16]
36. Masi G, Cupini S, Marcucci L, et al. Treatment with 5-fluorouracil/folinic acid, oxaliplatin, and irinotecan enables surgical resection of metastases in patients with initially unresectable metastatic colorectal cancer. *Ann Surg Oncol* 2006;13(1):58-65. doi: 10.1245/ASO.2006.03.094 [published Online First: 2005/12/24]
37. Nordlinger B, Sorbye H, Glimelius B, et al. Perioperative chemotherapy with FOLFOX4 and surgery versus surgery alone for resectable liver metastases from colorectal cancer (EORTC Intergroup trial 40983): a randomised controlled trial. *Lancet* 2008;371(9617):1007-16. doi: 10.1016/S0140-6736(08)60455-9 [published Online First: 2008/03/25]
38. Zeiss J, Merrick HW, Savolaine ER, et al. Fatty liver change as a result of hepatic artery infusion chemotherapy. *Am J Clin Oncol* 1990;13(2):156-60. [published Online First: 1990/04/01]
39. Moertel CG, Fleming TR, Macdonald JS, et al. Hepatic toxicity associated with fluorouracil plus levamisole adjuvant therapy. *J Clin Oncol* 1993;11(12):2386-90. doi: 10.1200/JCO.1993.11.12.2386 [published Online First: 1993/12/01]
40. Sorensen P, Edal AL, Madsen EL, et al. Reversible hepatic steatosis in patients treated with interferon alfa-2a and 5-fluorouracil. *Cancer* 1995;75(10):2592-6. [published Online First: 1995/05/15]
41. Peppercorn PD, Reznek RH, Wilson P, et al. Demonstration of hepatic steatosis by computerized tomography in patients receiving 5-fluorouracil-based therapy for advanced colorectal cancer. *Br J Cancer* 1998;77(11):2008-11. [published Online First: 1998/07/17]
42. Behrns KE, Tsiotos GG, DeSouza NF, et al. Hepatic steatosis as a potential risk factor for major hepatic resection. *J Gastrointest Surg* 1998;2(3):292-8. [published Online First: 1998/12/08]
43. Belghiti J, Hiramatsu K, Benoist S, et al. Seven hundred forty-seven hepatectomies in the 1990s: an update to evaluate the actual risk of liver resection. *J Am Coll Surg* 2000;191(1):38-46. [published Online First: 2000/07/18]
44. Parikh AA, Gentner B, Wu TT, et al. Perioperative complications in patients undergoing major liver resection with or without neoadjuvant chemotherapy. *J Gastrointest Surg* 2003;7(8):1082-8. [published Online First: 2003/12/17]
45. Kooby DA, Fong Y, Suriawinata A, et al. Impact of steatosis on perioperative outcome following hepatic resection. *J Gastrointest Surg* 2003;7(8):1034-44. [published Online First: 2003/12/17]
46. Rubbia-Brandt L, Audard V, Sartoretti P, et al. Severe hepatic sinusoidal obstruction associated with oxaliplatin-based chemotherapy in patients with metastatic colorectal cancer. *Ann Oncol* 2004;15(3):460-6. [published Online First: 2004/03/05]
47. Karoui M, Penna C, Amin-Hashem M, et al. Influence of preoperative chemotherapy on the risk of major hepatectomy for colorectal liver metastases. *Ann Surg* 2006;243(1):1-7. [published Online First: 2005/12/24]
48. Vauthey JN, Pawlik TM, Ribero D, et al. Chemotherapy regimen predicts steatohepatitis and an increase in 90-day mortality after surgery for hepatic colorectal metastases. *J Clin Oncol* 2006;24(13):2065-72. doi: 10.1200/jco.2005.05.3074 [published Online First: 2006/05/02]

For peer review only - http://bmjopen.bmj.com/site/about/guidelines.xhtml

49. Pawlik TM, Olino K, Gleisner AL, et al. Preoperative chemotherapy for colorectal liver metastases: impact on hepatic histology and postoperative outcome. *J Gastrointest Surg* 2007;11(7):860-8. doi: 10.1007/s11605-007-0149-4 [published Online First: 2007/05/12]
50. Hewes JC, Dighe S, Morris RW, et al. Preoperative chemotherapy and the outcome of liver resection for colorectal metastases. *World J Surg* 2007;31(2):353-64; discussion 65-6. doi: 10.1007/s00268-006-0103-8 [published Online First: 2007/01/16]
51. Nakano H, Oussoultzoglou E, Rosso E, et al. Sinusoidal injury increases morbidity after major hepatectomy in patients with colorectal liver metastases receiving preoperative chemotherapy. *Ann Surg* 2008;247(1):118-24. doi: 10.1097/SLA.0b013e31815774de [published Online First: 2007/12/25]
52. Hewes J. Re: Preoperative chemotherapy and the outcome of liver resection for colorectal metastases. *World J Surg* 2007;31(9):1902; author reply 03. doi: 10.1007/s00268-007-9065-8 [published Online First: 2007/05/10]
53. Soubrane O, Brouquet A, Zalinski S, et al. Predicting high grade lesions of sinusoidal obstruction syndrome related to oxaliplatin-based chemotherapy for colorectal liver metastases: correlation with post-hepatectomy outcome. *Ann Surg* 2010;251(3):454-60. doi: 10.1097/SLA.0b013e3181c79403 [published Online First: 2010/02/18]
54. Tamandl D, Klinger M, Eipeldauer S, et al. Sinusoidal obstruction syndrome impairs long-term outcome of colorectal liver metastases treated with resection after neoadjuvant chemotherapy. *Ann Surg Oncol* 2011;18(2):421-30. doi: 10.1245/s10434-010-1317-4 [published Online First: 2010/09/17]
55. Wolf PS, Park JO, Bao F, et al. Preoperative chemotherapy and the risk of hepatotoxicity and morbidity after liver resection for metastatic colorectal cancer: a single institution experience. *J Am Coll Surg* 2013;216(1):41-9. doi: 10.1016/j.jamcollsurg.2012.08.030 [published Online First: 2012/10/09]
56. Parkin E, O'Reilly DA, Adam R, et al. Equivalent survival in patients with and without steatosis undergoing resection for colorectal liver metastases following pre-operative chemotherapy. *Eur J Surg Oncol* 2014 doi: 10.1016/j.ejso.2014.07.040 [published Online First: 2014/09/06]
57. Vigano L, Capussotti L, De Rosa G, et al. Liver resection for colorectal metastases after chemotherapy: impact of chemotherapy-related liver injuries, pathological tumor response, and micrometastases on long-term survival. *Ann Surg* 2013;258(5):731-40; discussion 41-2. doi: 10.1097/SLA.0b013e3182a6183e [published Online First: 2013/09/21]
58. Vigano L, De Rosa G, Toso C, et al. Reversibility of chemotherapy-related liver injury. *J Hepatol* 2017;67(1):84-91. doi: 10.1016/j.jhep.2017.02.031 [published Online First: 2017/03/13]
59. Welsh FK, Tilney HS, Tekkis PP, et al. Safe liver resection following chemotherapy for colorectal metastases is a matter of timing. *Br J Cancer* 2007;96(7):1037-42. doi: 10.1038/sj.bjc.6603670 [published Online First: 2007/03/14]
60. Poon RT, Fan ST, Lo CM, et al. Extended hepatic resection for hepatocellular carcinoma in patients with cirrhosis: is it justified? *Ann Surg* 2002;236(5):602-11. doi: 10.1097/01.SLA.0000033038.38956.5E [published Online First: 2002/11/01]
61. Farges O, Malassagne B, Flejou JF, et al. Risk of major liver resection in patients with underlying chronic liver disease: a reappraisal. *Ann Surg* 1999;229(2):210-5. [published Online First: 1999/02/19]
62. Ryan P, Nanji S, Pollett A, et al. Chemotherapy-induced liver injury in metastatic colorectal cancer: semiquantitative histologic analysis of 334 resected liver specimens shows that vascular injury but not steatohepatitis is associated with preoperative chemotherapy. *Am J Surg Pathol* 2010;34(6):784-91. doi: 10.1097/PAS.0b013e3181dc242c [published Online First: 2010/04/28]
63. Stockmann M, Lock JF, Malinowski M, et al. The LiMAx test: a new liver function test for predicting postoperative outcome in liver surgery. *HPB (Oxford)* 2010;12(2):139-46. doi: 10.1111/j.1477-2574.2009.00151.x [published Online First: 2010/05/25]

For peer review only - http://bmjopen.bmj.com/site/about/guidelines.xhtml

64. Jiang Z, Dragin N, Jorge-Nebert LF, et al. Search for an association between the human CYP1A2 genotype and CYP1A2 metabolic phenotype. *Pharmacogenet Genomics* 2006;16(5):359-67. doi: 10.1097/01.fpc.0000204994.99429.46 [published Online First: 2006/04/13]
65. Palmer CN, Coates PJ, Davies SE, et al. Localization of cytochrome P-450 gene expression in normal and diseased human liver by in situ hybridization of wax-embedded archival material. *Hepatology* 1992;16(3):682-7. [published Online First: 1992/09/01]
66. George J, Murray M, Byth K, et al. Differential alterations of cytochrome P450 proteins in livers from patients with severe chronic liver disease. *Hepatology* 1995;21(1):120-8. [published Online First: 1995/01/01]
67. Schoeller DA, Schneider JF, Solomons NW, et al. Clinical diagnosis with the stable isotope 13C in CO2 breath tests: methodology and fundamental considerations. *J Lab Clin Med* 1977;90(3):412-21. [published Online First: 1977/09/01]
68. Stockmann M, Lock JF, Riecke B, et al. Prediction of postoperative outcome after hepatectomy with a new bedside test for maximal liver function capacity. *Ann Surg* 2009;250(1):119-25. doi: 10.1097/SLA.0b013e3181ad85b5 [published Online First: 2009/06/30]
69. Lock JF, Malinowski M, Seehofer D, et al. Function and volume recovery after partial hepatectomy: influence of preoperative liver function, residual liver volume, and obesity. *Langenbecks Arch Surg* 2012;397(8):1297-304. doi: 10.1007/s00423-012-0972-2 [published Online First: 2012/06/26]
70. Jara M, Reese T, Malinowski M, et al. Reductions in post-hepatectomy liver failure and related mortality after implementation of the LiMAx algorithm in preoperative work-up: a single-centre analysis of 1170 hepatectomies of one or more segments. *HPB (Oxford)* 2015;17(7):651-8. doi: 10.1111/hpb.12424 [published Online First: 2015/06/11]
71. Jara M, Bednarsch J, Valle E, et al. Reliable assessment of liver function using LiMAx. *J Surg Res* 2015;193(1):184-9. doi: 10.1016/j.jss.2014.07.041 [published Online First: 2014/08/26]
72. Jara M, Bednarsch J, Malinowski M, et al. Effects of oxaliplatin-based chemotherapy on liver function--an analysis of impact and functional recovery using the LiMAx test. *Langenbecks Arch Surg* 2016;401(1):33-41. doi: 10.1007/s00423-015-1352-5 [published Online First: 2015/10/29]
73. Lock JF, Westphal T, Rubin T, et al. LiMAx Test Improves Diagnosis of Chemotherapy-Associated Liver Injury Before Resection of Colorectal Liver Metastases. *Ann Surg Oncol* 2017;24(9):2447-55. doi: 10.1245/s10434-017-5887-2 [published Online First: 2017/05/19]
74. Foster KJ, Dewbury KC, Griffith AH, et al. The accuracy of ultrasound in the detection of fatty infiltration of the liver. *Br J Radiol* 1980;53(629):440-2. doi: 10.1259/0007-1285-53-629-440 [published Online First: 1980/05/01]
75. Bydder GM, Kreel L, Chapman RW, et al. Accuracy of computed tomography in diagnosis of fatty liver. *Br Med J* 1980;281(6247):1042. [published Online First: 1980/10/18]
76. Piekarski J, Goldberg HI, Royal SA, et al. Difference between liver and spleen CT numbers in the normal adult: its usefulness in predicting the presence of diffuse liver disease. *Radiology* 1980;137(3):727-9. doi: 10.1148/radiology.137.3.6934563 [published Online First: 1980/12/01]
77. Meek DR, Mills PR, Gray HW, et al. A comparison of computed tomography, ultrasound and scintigraphy in the diagnosis of alcoholic liver disease. *Br J Radiol* 1984;57(673):23-7. doi: 10.1259/0007-1285-57-673-23 [published Online First: 1984/01/01]
78. Graif M, Yanuka M, Baraz M, et al. Quantitative estimation of attenuation in ultrasound video images: correlation with histology in diffuse liver disease. *Invest Radiol* 2000;35(5):319-24. [published Online First: 2000/05/10]

For peer review only - http://bmjopen.bmj.com/site/about/guidelines.xhtml

79. Reeder SB, Cruite I, Hamilton G, et al. Quantitative Assessment of Liver Fat with Magnetic Resonance Imaging and Spectroscopy. *J Magn Reson Imaging* 2011;34(4):spcone. doi: 10.1002/jmri.22775 [published Online First: 2011/10/26]
80. Davies RJ, Saverymuttu SH, Fallowfield M, et al. Paradoxical lack of ultrasound attenuation with gross fatty change in the liver. *Clin Radiol* 1991;43(6):393-6. [published Online First: 1991/06/01]
81. Thomsen C, Becker U, Winkler K, et al. Quantification of liver fat using magnetic resonance spectroscopy. *Magn Reson Imaging* 1994;12(3):487-95. [published Online First: 1994/01/01]
82. Hatfield MK, Beres RA, Sane SS, et al. Percutaneous imaging-guided solid organ core needle biopsy: coaxial versus noncoaxial method. *AJR Am J Roentgenol* 2008;190(2):413-7. doi: 10.2214/AJR.07.2676 [published Online First: 2008/01/24]
83. Ratziu V, Charlotte F, Heurtier A, et al. Sampling variability of liver biopsy in nonalcoholic fatty liver disease. *Gastroenterology* 2005;128(7):1898-906. [published Online First: 2005/06/09]
84. El-Badry AM, Breitenstein S, Jochum W, et al. Assessment of hepatic steatosis by expert pathologists: the end of a gold standard. *Ann Surg* 2009;250(5):691-7. doi: 10.1097/SLA.0b013e3181bcd6dd [published Online First: 2009/10/07]
85. Bravo AA, Sheth SG, Chopra S. Liver biopsy. *N Engl J Med* 2001;344(7):495-500. doi: 10.1056/NEJM200102153440706 [published Online First: 2001/02/15]
86. Haaga JR. Magnetic resonance imaging of the liver. *Radiol Clin North Am* 1984;22(4):879-90. [published Online First: 1984/12/01]
87. Stark DD, Moss AA, Goldberg HI. Nuclear magnetic resonance of the liver, spleen, and pancreas. *Cardiovasc Intervent Radiol* 1986;8(5-6):329-41. [published Online First: 1986/01/01]
88. Kanzer GK, Weinreb JC. Magnetic resonance imaging of diseases of the liver and biliary system. *Radiol Clin North Am* 1991;29(6):1259-84. [published Online First: 1991/11/01]
89. Lee JK, Dixon WT, Ling D, et al. Fatty infiltration of the liver: demonstration by proton spectroscopic imaging. Preliminary observations. *Radiology* 1984;153(1):195-201. doi: 10.1148/radiology.153.1.6089264 [published Online First: 1984/10/01]
90. Kukuk GM, Hittatiya K, Sprinkart AM, et al. Comparison between modified Dixon MRI techniques, MR spectroscopic relaxometry, and different histologic quantification methods in the assessment of hepatic steatosis. *Eur Radiol* 2015;25(10):2869-79. doi: 10.1007/s00330-015-3703-6 [published Online First: 2015/04/24]
91. Szczepaniak LS, Nurenberg P, Leonard D, et al. Magnetic resonance spectroscopy to measure hepatic triglyceride content: prevalence of hepatic steatosis in the general population. *Am J Physiol Endocrinol Metab* 2005;288(2):E462-8. doi: 10.1152/ajpendo.00064.2004 [published Online First: 2004/09/02]
92. Clark JM, Brancati FL, Diehl AM. Nonalcoholic fatty liver disease. *Gastroenterology* 2002;122(6):1649-57. [published Online First: 2002/05/23]
93. Szczepaniak LS, Babcock EE, Schick F, et al. Measurement of intracellular triglyceride stores by H spectroscopy: validation in vivo. *Am J Physiol* 1999;276(5 Pt 1):E977-89. [published Online First: 1999/05/18]
94. Bohte AE, van Werven JR, Bipat S, et al. The diagnostic accuracy of US, CT, MRI and 1H-MRS for the evaluation of hepatic steatosis compared with liver biopsy: a meta-analysis. *Eur Radiol* 2011;21(1):87-97. doi: 10.1007/s00330-010-1905-5 [published Online First: 2010/08/04]
95. Banerjee R, Pavlides M, Tunnicliffe EM, et al. Multiparametric magnetic resonance for the non-invasive diagnosis of liver disease. *J Hepatol* 2014;60(1):69-77. doi: 10.1016/j.jhep.2013.09.002 [published Online First: 2013/09/17]
96. McPherson S, Jonsson JR, Cowin GJ, et al. Magnetic resonance imaging and spectroscopy accurately estimate the severity of steatosis provided the stage of fibrosis is considered. *J Hepatol* 2009;51(2):389-97. doi: 10.1016/j.jhep.2009.04.012 [published Online First: 2009/06/10]

For peer review only - http://bmjopen.bmj.com/site/about/guidelines.xhtml

97. Valls C, Iannacconne R, Alba E, et al. Fat in the liver: diagnosis and characterization. *Eur Radiol* 2006;16(10):2292-308. doi: 10.1007/s00330-006-0146-0 [published Online First: 2006/02/16]
98. van Werven JR, Hoogduin JM, Nederveen AJ, et al. Reproducibility of 3.0 Tesla magnetic resonance spectroscopy for measuring hepatic fat content. *J Magn Reson Imaging* 2009;30(2):444-8. doi: 10.1002/jmri.21837 [published Online First: 2009/07/25]
99. Longo R, Pollesello P, Ricci C, et al. Proton MR spectroscopy in quantitative in vivo determination of fat content in human liver steatosis. *J Magn Reson Imaging* 1995;5(3):281-5. [published Online First: 1995/05/01]
100. Longo R, Ricci C, Masutti F, et al. Fatty infiltration of the liver. Quantification by 1H localized magnetic resonance spectroscopy and comparison with computed tomography. *Invest Radiol* 1993;28(4):297-302. [published Online First: 1993/04/01]
101. Idilman IS, Aniktar H, Idilman R, et al. Hepatic steatosis: quantification by proton density fat fraction with MR imaging versus liver biopsy. *Radiology* 2013;267(3):767-75. doi: 10.1148/radiol.13121360 [published Online First: 2013/02/06]
102. Tang A, Tan J, Sun M, et al. Nonalcoholic fatty liver disease: MR imaging of liver proton density fat fraction to assess hepatic steatosis. *Radiology* 2013;267(2):422-31. doi: 10.1148/radiol.12120896 [published Online First: 2013/02/06]
103. Noureddin M, Lam J, Peterson MR, et al. Utility of magnetic resonance imaging versus histology for quantifying changes in liver fat in nonalcoholic fatty liver disease trials. *Hepatology* 2013;58(6):1930-40. doi: 10.1002/hep.26455 [published Online First: 2013/05/23]
104. Parkin E, O'Reilly DA, Plumb AA, et al. Digital histology quantification of intra-hepatic fat in patients undergoing liver resection. *Eur J Surg Oncol* 2015;41(8):1020-7. doi: 10.1016/j.ejso.2015.05.003 [published Online First: 2015/06/10]
105. Coe P, Williams S, Morris D, et al. Development of MR quantified pancreatic fat deposition as a cancer risk biomarker (PanORAMA) [submitted]. *Pancreatology* 2018

For peer review only

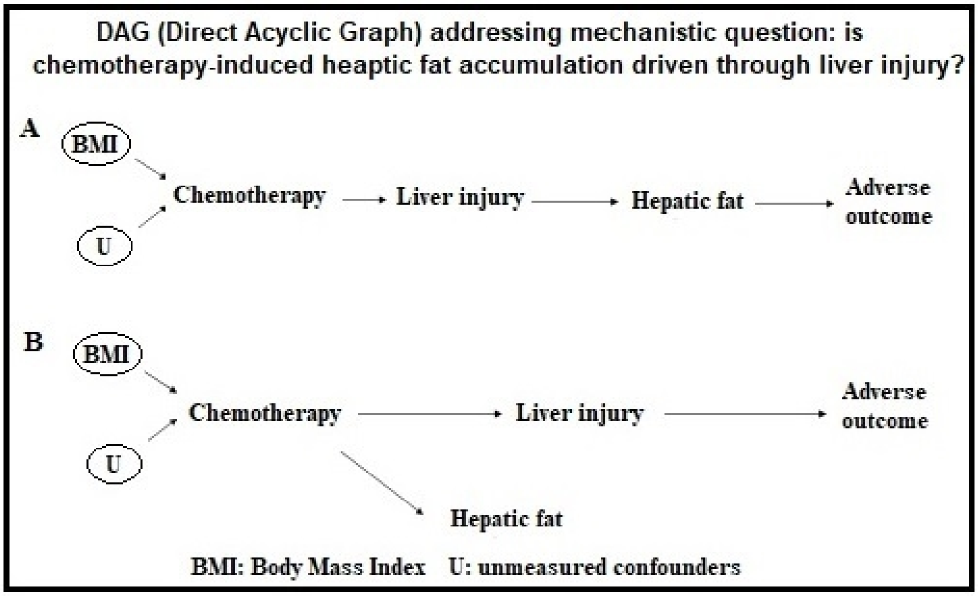

Direct Acyclic Graph

43x27mm (300 x 300 DPI)

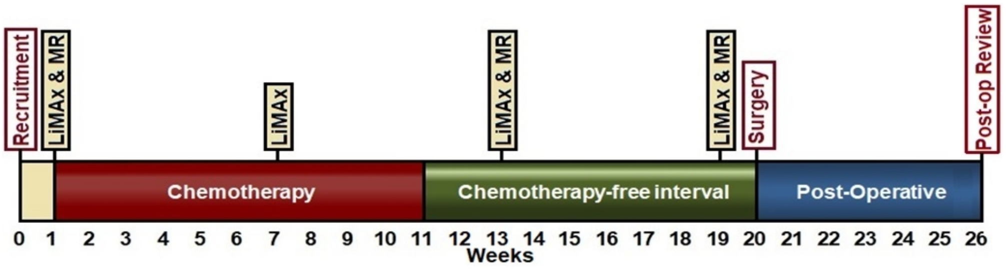

Study Participant Timeline

87x23mm (300 x 300 DPI)

APPENDIX 1 - STUDY SCHEDULE

				Standard care assessments		Experimental added assessments		
**Timeline (weeks)**		**Oncology Outpatient Department**	**Blood Tests**	**CT**	**Histology**	**LiMAx**	**MR**	**EQ-5D**
**BASELINE**		**Patient identification HPB MDT/ Oncology**		✓				
		**New Patient Oncology Clinic – CLiFF Study information**	✓					
**1**	Day 1	Oncology Clinic – Assessment, Bloods	✓			✓	✓	✓
	Day 2	**Chemotherapy- Cycle 1**						
**3**	Day 1	Oncology Clinic – Assessment, Bloods	✓					
	Day 2	**Chemotherapy- Cycle 2**						
**5**	Day 1	Oncology Clinic – Assessment, Bloods	✓					
	Day 2	**Chemotherapy- Cycle 3**						
**7**	Day 1	Oncology Clinic – Assessment, Bloods	✓			✓		
	Day 2	**Chemotherapy- Cycle 4**						
**9**	Day 1	Oncology Clinic – Assessment, Bloods	✓					
	Day 2	**Chemotherapy- Cycle 5**						
**11**	Day 1	Oncology Clinic – Assessment, Bloods	✓					
	Day 2	**Chemotherapy- Cycle 6**						
**13**	*Chemo finished	Oncology Clinic – Final Assessment, Bloods **ONCOLOGY DISCHARGE**	✓	✓		✓	✓	✓
**19**	**Post-Chemotherapy Week 8**							
	**Additional Visit to Christie Site** LiMAx and MR					✓	✓	✓
**Pre-Op** (~ Wk 19/20)		Pre-operative assessment clinic at Manchester Foundation Trust HPB Dept	✓					
**6 weeks post-op** (~ Wk 26)		Post-operative review clinic at Manchester Foundation Trust HPB Dept	✓		✓			✓

APPENDIX 2 – STUDY ASSESSMENTS

	Event	Baseline	Pre-chemo	2nd cycle	3rd cycle	4th cycle	5th cycle	6th cycle	Following last cycle of chemotherapy	Approx 8 wks post-chemo/ pre-op assessment	Post-op Assessment
**Standard Care Events/ Assessments**	History	✓									
	Physical examination	✓	✓	✓	✓	✓	✓	✓	✓	✓	✓
	Height	✓									
	Weight	✓	✓	✓	✓	✓	✓	✓	✓	✓	✓
	WHO perf score	✓	✓	✓	✓	✓	✓	✓	✓	✓	✓
	FBC	✓	✓	✓	✓	✓	✓	✓	✓	✓	✓
	Biochemistry [b]	✓	✓	✓	✓	✓	✓	✓	✓	✓	✓
	CEA	✓								✓	
	Coagulation [c]	✓	✓	✓	✓	✓	✓	✓	✓	✓	
	Pregnancy test [d]	✓									
	CT	✓							✓		
	Histopathology										✓
**Experimental Additional Events/ Assessments**	Informed Consent		✓								
	EQ-5D Score		✓						✓	✓	✓
	MR		✓						✓	✓	
	LiMAx		✓			✓			✓	✓	

For peer review only - http://bmjopen.bmj.com/site/about/guidelines.xhtml
